# Mechanistic basis of an epistatic interaction reducing age at onset in hereditary spastic paraplegia

**DOI:** 10.1093/brain/awy034

**Published:** 2018-02-22

**Authors:** Timothy Newton, Rachel Allison, James R Edgar, Jennifer H Lumb, Catherine E Rodger, Paul T Manna, Tania Rizo, Zacharias Kohl, Anders O H Nygren, Larissa Arning, Rebecca Schüle, Christel Depienne, Lisa Goldberg, Christiane Frahm, Giovanni Stevanin, Alexandra Durr, Ludger Schöls, Beate Winner, Christian Beetz, Evan Reid

**Affiliations:** 1 Department of Medical Genetics and Cambridge Institute for Medical Research, University of Cambridge, UK; 2 Department of Clinical Biochemistry and Cambridge Institute for Medical Research, University of Cambridge, Cambridge CB2 0XY, UK; 3 Department of Stem Cell Biology, Friedrich-Alexander University Erlangen-Nuernberg (FAU), Erlangen, Germany; 4 Department of Molecular Neurology, Friedrich-Alexander University Erlangen-Nuernberg (FAU), Erlangen, Germany; 5 MRC-Holland, Amsterdam, The Netherlands; 6 Department of Human Genetics, Ruhr-University, Bochum, Germany; 7 Center for Neurology and Hertie Institute for Clinical Brain Research, Eberhard-Karls-University, 72076 Tübingen, Germany; 8 German Center of Neurodegenerative Diseases (DZNE), 72076 Tübingen, Germany; 9 ICM Brain and Spine Institute, INSERM U1127, CNRS UMR7225, Sorbonne Universites, UPMC Univ Paris VI UMR_S1127, Paris, France; 10 APHP, Genetic Department, Pitie-Salpêtrière University Hospital, Paris, France; 11 Department of Clinical Chemistry and Laboratory Diagnostics, Jena University Hospital, Jena, Germany; 12 Hans Berger Department of Neurology, Jena University Hospital, Jena, Germany; 13 Ecole Pratique des Hautes Etudes, PSL Research University, Paris, France

**Keywords:** axonopathy, histone methyltransferase, epistasis, endosomal tubule fission, lysosome

## Abstract

Many genetic neurological disorders exhibit variable expression within affected families, often exemplified by variations in disease age at onset. Epistatic effects (i.e. effects of modifier genes on the disease gene) may underlie this variation, but the mechanistic basis for such epistatic interactions is rarely understood. Here we report a novel epistatic interaction between *SPAST* and the contiguous gene *DPY30*, which modifies age at onset in hereditary spastic paraplegia, a genetic axonopathy. We found that patients with hereditary spastic paraplegia caused by genomic deletions of *SPAST* that extended into *DPY30* had a significantly younger age at onset. We show that, like spastin, the protein encoded by *SPAST*, the DPY30 protein controls endosomal tubule fission, traffic of mannose 6-phosphate receptors from endosomes to the Golgi, and lysosomal ultrastructural morphology. We propose that additive effects on this pathway explain the reduced age at onset of hereditary spastic paraplegia in patients who are haploinsufficient for both genes.

## Teaser

The mechanisms underlying disease modifier gene effects are rarely understood. Newton *et al.* report that deletion of *DPY30* reduces age at onset in hereditary spastic paraplegia caused by *SPAST* mutations. They demonstrate that both genes regulate cellular pathways that pathologically impact lysosome function, providing a mechanistic explanation for this interaction.

## Introduction

A hallmark of many genetic neurodegenerative disorders is variable expression within affected families, often exemplified by variations in disease age at onset. In some diseases, typically those caused by triplet repeat expansions, this variability is explained by the dynamic nature of the mutation (Orr and Zoghbi, 2007). However, in conditions where the pathogenic mutation is static, epistatic effects, where alleles at other genes modify the disease phenotype, are often suggested as an explanation. The underlying biological basis for such epistatic effects may involve additive deleterious effects on cellular pathways in which the proteins encoded by the disease and modifier gene both participate. However, in human single gene disease in general, and in neurodegenerative disease in particular, there are few examples where the underlying functional basis for such an epistatic effect has been explained.

The hereditary spastic paraplegias (HSPs) are a group of single gene disorders in which the main pathological feature is a dying-back axonopathy involving the longest corticospinal tract axons (Harding, 1993; Reid, 1999; Fink, 2006). Although HSP is caused by mutations in many genes (Hensiek *et al.*, 2015), mutations in *SPAST* (also known as SPG4) are by far the most frequent, affecting up to 60% of families with autosomal dominant HSP, and around 10–20% of cases unselected by family history (Hazan *et al.*, 1999; Fonknechten *et al.*, 2000; Sauter *et al.*, 2002; Schule *et al.*, 2016).

*SPAST* encodes the microtubule severing ATPase enzyme spastin, which uses the energy of ATP hydrolysis to create internal breaks within microtubules (Errico *et al.*, 2002; Evans *et al.*, 2005; Roll-Mecak and Vale, 2008; Lumb *et al.*, 2012). Spastin localizes to sites of contact between the endoplasmic reticulum and endosomes, and we reported recently that it is required for efficient fission of endosomal tubules from the endosome, which occurs specifically at these contact sites (Allison *et al.*, 2013, 2017). Defective tubule fission in cells lacking spastin manifests as an increase in the number and length of tubules attached to the endosomal body. This is accompanied by defective sorting of receptors that traffic away from endosomes via this tubular compartment, including of mannose 6-phosphate receptors (M6PRs) (Allison *et al.*, 2013, 2017). M6PRs normally cycle between endosomes and the trans Golgi network; at the trans Golgi network they capture M6P-tagged lysosomal enzymes for delivery back to the endolysosomal degradative compartment. Inhibition of M6PR traffic away from the endosome in cells lacking spastin causes accumulation of M6PRs in the endolysosomal compartment and reduced availability at the trans Golgi network, resulting in diminished capture of lysosomal enzymes. This causes mistrafficking of lysosomal enzymes and a failure of enzyme delivery to endolysosomes, which in turn causes the development of abnormally enlarged lysosomes with characteristic ultrastructural morphology in spastin-HSP cellular models. Importantly, this includes primary neurons from a spastin knock-in mouse expressing a disease-associated spastin ATPase domain mutation, and induced pluripotent stem cell (iPSC)-derived spastin-HSP patient neurons (Connell *et al.*, 2016; Allison *et al.*, 2017). As a similar lysosomal abnormality has been observed in several other subtypes of HSP, we have proposed that it represents a common pathological mechanism for the disease (Khundadze *et al.*, 2013; Renvoise *et al.*, 2014; Hirst *et al.*, 2015; Varga *et al.*, 2015; Allison *et al.*, 2017).

Spastin-HSP shows considerable variation in age at onset, both within and between families. This can range from early childhood to late adult life (Fonknechten *et al.*, 2000; Lindsey *et al.*, 2000). The *SPAST* mutational spectrum is broad, incorporating nonsense, frameshift, splice site and missense mutations, strongly suggesting that the disease mechanism is haploinsufficiency in most, if not all, cases (Fonknechten *et al.*, 2000). With regard to these small genetic alterations, to date there is no unambiguous genotype–phenotype correlation related to age at onset of disease, although the presence of a hypomorphic variant (S44L) in combination with a disease causing mutation reduces age at onset (Yip *et al.*, 2003; Svenson *et al.*, 2004; Shoukier *et al.*, 2009).

The contribution of large genomic rearrangements to the *SPAST* mutational spectrum was recognized by studies that used cDNA analysis and Southern blotting to identify single or multi-exon deletions (Sauter *et al.*, 2002; Iwanaga *et al.*, 2005). Subsequent development of a *SPAST*-targeted multiplex ligation-dependent probe amplification (MLPA) assay revealed in two studies that large genomic abnormalities account for up to 20% of disease-associated *SPAST* mutations (Beetz *et al.*, 2006; Depienne *et al.*, 2007). These initial MLPA studies compared clinical characteristics of deletion carriers versus carriers of conventional ‘small’ mutations and in one an earlier age at onset in *SPAST* deletion-positive patients was found (Depienne *et al.*, 2007). Numerous reports on *SPAST* rearrangements have been published since (see Human Gene Mutation Database website: http://www.hgmd.cf.ac.uk/ac/index.php), and the association of large deletions with reduced age at onset was again observed (Chelban *et al.*, 2017). The question of how different mutational classes, which all likely act via a haploinsufficiency mechanism, could have differing clinical outcomes has not been resolved.

In this study we confirm that exonic deletion of *SPAST* is associated with a significant reduction in age at onset of HSP, but show that this reduction is accounted for by a subset of patients in whom the deletion extends into the contiguous gene *DPY30*. The protein encoded by *DPY30* has been implicated in endosome-to-Golgi traffic (Xu *et al.*, 2009). We therefore investigated whether the DPY30 protein regulates similar endosomal trafficking steps to spastin. We found that cultured cells lacking DPY30 had increased endosomal tubulation, defective traffic of M6PR from endosomes to the Golgi apparatus, and abnormal lysosomal ultrastructural appearances that were highly similar to those seen in spastin-HSP models. Thus we propose that haploinsufficiency of spastin and DPY30 causes reduced age at onset in HSP via additive deleterious effects on endosomal membrane traffic and consequent lysosomal function. This study provides support for the general hypothesis that epistasis can be caused by additive effects on cellular pathways in which the genes involved participate.

## Materials and methods

### Patients and ethical approval

#### Subjects who donated fibroblast lines

The patients included were of western European ethnic origin with typical characteristics of pure HSP and mutations in *SPAST*, as summarized below. The control was an unrelated healthy subject from the same ethnic background, with no history of movement disorder or neurological disease. Human fibroblasts were obtained from dermal punch biopsies from the upper arm, following Institutional Review Board approval (Nr. 4120: Generierung von humanen neuronalen Modellen bei neurodegenerativen Erkrankungen) and written informed consent at the movement disorder clinic at the Department of Molecular Neurology, Universitätsklinikum Erlangen (Erlangen, Germany). Further details of the patients and cell lines derived from them are as follows:

Patient 1 was a 51-year-old male with *SPAST* deletion involving exons 2–9, with age at onset 39 years. Patient 2 was a 50-year-old female with mutation involving deletion of exons 1–7 of *SPAST* and exons 1–5 of DPY30, who had an age at onset below 20 years of age.

The control was a healthy 41-year-old male subject. Fibroblast were designated UKERf1JF-X-001 from the control subject, UKERf29U-X-001 from Patient 1 and UKERfVRV-X-001 from Patient 2.

#### Patients who participated in genetic studies

Samples were obtained from peripheral blood samples from patients with clinically pure HSP, obtained after informed written consent. Age at onset information was available for all included patients. Where required, the research was performed according to protocols reviewed and approved by local institutional review boards (IRBs); Cambridge Local Research Ethics Committee, Cambridge, UK (LREC 96/268); Paris-Necker ethics committee (approval RBM 01-29 and RBM 03-48 to A.D.; patients gave informed consent and blood samples were collected in accordance with local French regulations); the ethics committee of the Medical Faculty of Tübingen University (GeNeMove study; approval 277/2004).

### Antibodies

Rabbit polyclonal DPY30, RBBP5, ASH2L and BIG1 antibodies were from Bethyl Laboratories. Rabbit polyclonal anti-GFP (6556) and mouse monoclonal anti-M6PR (ab2733) were from Abcam. Mouse monoclonal anti-Snx1 was obtained from BD Transduction Laboratories. Mouse monoclonal anti-LC3 was obtained from MBL (clone 4E12, catalogue number M152-3). Rabbit polyclonal anti-spastin 86-340 and CD8 antibodies were produced as previously described (Seaman, 2004; Connell *et al.*, 2009). Mouse monoclonal anti-LAMP1 (H4A3) was obtained from Santa Cruz Biotechnology. Rabbit polyclonal anti-GAPDH (2118) was from Cell Signalling Technology. Alexa Fluor® 488 and 568 labelled secondary antibodies for immunofluorescence were obtained from Molecular Probes. Peroxidase conjugated secondary antibodies for western blotting were obtained from Sigma.

### Stable cell lines

A HeLa cell line stably expressing GFP-GOLPH3 and CD8-CIMPR was a gift from Matthew Seaman (Seaman, 2004).

### Cell culture

HeLaM cells were maintained as previously described (Connell *et al.*, 2009). HeLaM cells stably expressing GFP-GOLPH3 and CD8-CIMPR were cultured in the presence of both 500 µg/ml Geneticin (Invitrogen) and 1 µg/ml Puromycin (Sigma-Aldrich). Human fibroblasts were cultured in Iscove’s modified Dulbecco’s medium containing 15% foetal bovine serum (FBS, Invitrogen) and 1× penicillin/streptomycin and passaged using TrypLE™ (all Invitrogen).

### MLPA assays

For MLPA we used probe mixes P165 and P211 (MRC-Holland, The Netherlands) according to the manufacturer’s recommendations. Analysis of raw MLPA data, and calculation of zygosity were performed as described previously (Beetz *et al.*, 2006).

### Reverse transcriptase-PCR

Fibroblasts were grown to ∼50% confluence, trypsinized, harvested, and homogenized in a glass-glass potter in order to isolate total RNA with the RNeasy® kit (Qiagen) following the manufacturer’s instructions. Quantitative real-time PCR was performed in a 20 µl amplification mixture consisting of Brilliant® II SYBR® Green QPCR Master Mix (Stratagene), cDNA (equivalent to 25 ng reverse-transcribed RNA) and primers (250 nM final concentration each; sequences available upon request). Transcripts were amplified with Rotor Gene 6000 (Corbett Life Science, now Qiagen). Expression levels were calculated using the Pfaffl equation (Pfaffl, 2001) and normalized against *GAPDH* and *HMBS*. To derive values for Patients 1 and 2, geometrical means from three runs were expressed as fractions of the control sample.

### Short interfering RNA transfection

Cells were transfected with the relevant siRNAs, using Oligofectamine™ transfection reagent (Invitrogen), according to a protocol modified from Connell *et al.* (2009). Briefly, cells were plated into a well of a 6-well plate and transfected after 24 h. Cells were harvested 48–96 h later. The efficiency of siRNA knock-down was verified by immunoblotting cell lysates and/or by immunoflourescence microscopy of fixed cells, with an antibody against the relevant protein. siRNA was used at a final concentration of 10 nM for each gene targeted. Target sequences and manufacturers for siRNA were as follows:

*SPAST*: *SPAST* 1: 5′-GAACUUCAACCUUCUAUAA (Dharmacon D-014070-01); *SPAST* 3: 5′-UAUAAGUGCUGCAAGUUUA (Dharmacon D-014070-03). *DPY30* 10: 5′-CAGACAACGUUGAGAGAAU (Dharmacon J-014923-10); *DPY30* 11: 5′-CCACCAAAUCCCAUUGAAU (Dharmacon J-014923-11); *DPY30* 12: 5′-UUUACAUGAUUAAGAGGCA (Dharmacon J-014923-12).

*ASH2L*: *ASH2L* 5: 5′-AAAGAUGGCUAUCGGUAUA (Dharmacon J-019831-05); *ASH2L* 6: 5′-GGACCAUGCUUCAAGUAUC (Dharmacon J-019831-06); *ASH2L* 8: 5′- GAGACAGAAUCAUCUAAUG (Dharmacon J-019831-08).

*RBBP5*: *RBBP5* 5: 5′-UAACACGGCAGAUCGAAUA (Dharmacon J-012008-05); *RBBP5* 6: 5′-UAUAGAACUUCAAGGAGUA (Dharmacon J-012008-06); *RBBP5* 8: 5′-GAUGGAACUUUGGAUUGUA (Dharmacon J-012008-08).

BIG1 (now known as *ARFGEF1*): *BIG1 5*: GUCCAAAUGUCCUCGCAUA (Dharmacon J-012207-05); *BIG1 6*: GAACAGGUCUCCAACAAUU (Dharmacon J-012207-05); *BIG1 7*: GAUCACAAAUGGAUGGUUA (Dharmacon J-012207-05); *BIG1 8*: GAUGAUAGAUUGUCAGUCU (Dharmacon J-012207-05).

### Immunofluorescence microscopy

Cells were fixed at room temperature in 3.8% (v/v) formaldehyde in phosphate-buffered saline (PBS) and permeabilized in PBS containing 0.1% (v/v) saponin (Sigma) or 0.1% (v/v) Triton™ X-100 (Sigma). Coverslips were labelled with primary and secondary antibodies as previously described (Connell *et al.*, 2009). Slides were analysed with a Zeiss LSM880 confocal microscope (100× NA 1.40 oil immersion objective, 37°C), or Zeiss AxioImager Z2 Motorized Upright Microscope (63× NA 1.40 oil immersion objective, room temperature, Zeiss Axiocam 506). Co-localization of immunofluorescence signals was quantified using Volocity Image Analysis Software. Images were subsequently processed using Adobe Photoshop and Illustrator and ZEN analysis software.

### Endosomal tubulation counts on fixed cells

Cells were processed for immunofluorescence microscopy and imaged with a Zeiss AxioImager Motorized Upright Microscope under a 63×/1.4 NA oil immersion objective as described above. Tubulation was quantified using one of two methods: (i) as described previously (Allison *et al.*, 2017), images of 100 cells per condition were randomized and the percentage of cells with at least one tubule longer than 2 µm was counted blind; or (ii) images were analysed computationally using a system described previously, which determined the number of tubules per cell, the mean length of the longest tubule in each cell and the percentage of cells containing a tubule (Newton and Reid, 2016).

### Cation independent M6PR trafficking assay

Following a 48-h siRNA knock-down of the relevant protein, HeLa cells stably expressing GFP-GOLPH3 and CD8-ciM6PR tail were subject to ciM6PR trafficking assays as described in Seaman (2007). Briefly, following incubation in medium containing anti-CD8 at 4°C, cells were incubated for 30 min at 37°C before fixation and processing for immunofluorescence microscopy with a Zeiss LSM880 confocal microscope as described above. Co-localization of appropriate proteins was quantified using Volocity Image Analysis Software.

#### Magic Red cathepsin B assays

Cells were subject to siRNA knock-down with the relevant siRNAs and incubated with transfection reagents for 96 h. Cells were harvested with trypsin, pelleted at 900 rpm for 3 min, and then resuspended in 250 µl Dulbecco’s modified Eagle medium (DMEM) supplemented with 10 µl of a 26× stock solution (in DMSO) of Magic Red™ cathepsin B (Bio-Rad) and incubated for 1 h at 37°C. Following the incubation cells were washed in PBS, resuspended in 500 µl DMEM and fluorescence at 561 nm analysed by fluorescence activated cell sorting (FACS) on a BD Fortessa analyser.

### Lysosome quantification

To determine the percentage of cells with large lysosomes, fixed cells labelled with LAMP1 were processed for immunofluorescence microscopy as described above, and imaged with a Zeiss AxioImager Motorized Upright Microscope as described above. One hundred cells were recorded per experimental condition. Images were randomized and the largest lysosome per cell measured using ZEN analysis software. Lysosomes were classified as ‘large’ if they exceeded 1.8 µm in diameter.

### Lysosome pH measurement

Lysosomal pH measurement was performed as described previously (Allison *et al.*, 2017). Briefly, HeLa cells were incubation for 4 h at 37°C in medium containing 1 mg/ml dextran conjugated to Oregon Green® (pH sensitive) or tetramethylrhodamine (pH insensitive). This was followed by a 20-h unlabelled chase before imaging with live cell microscopy at 37°C using a Zeiss LSM780 microscope (63× NA 1.40 oil objective). Individual puncta were identified with ImageJ and the ratio of Oregon Green® to tetramethylrhodamine signal was quantified for each punctum. The pH of each punctum was determined against a standard curve of fluorescence ratio, generated using cells incubated with 10 µm nigericin and 5 µm monensin in buffers ranging from pH 4.5 to 6.5.

### Electron microscopy

HeLa cells were grown on Thermanox® (Nunc) plastic coverslips and fixed with 2% paraformaldehyde, 2.5% glutaraldehyde, 0.1 M cacodylate buffer (pH 7.2). Samples were post-fixed with 1% osmium tetroxide: 1.5% potassium ferricyanide before being incubated with 1% tannic acid to enhance contrast. Cells were dehydrated using increasing percentages of ethanol before being embedded onto EPON stubs. Resin was cured overnight at 65°C and coverslips were removed using a heat block. Ultrathin (50–70 nm) conventional sections were cut using a diamond knife mounted to a Reichert Ultracut S ultramicrotome. Sections were collected onto copper grids. Grids were stained using lead citrate. Sections were viewed on a FEI Tecnai transmission electron microscope at a working voltage of 80 kV.

### Statistical analysis

Statistical analyses were done with paired two-tailed (or in the case of the M6PR trafficking assay one-tailed) *t*-tests, using GraphPad Prism 5.01 for Windows (GraphPad Software, San Diego). Histograms show mean ± standard error of the mean (SEM).

## Results

### Decreased age at onset in carriers of *SPAST* deletions correlates with involvement of exon 1

**Figure 1:**
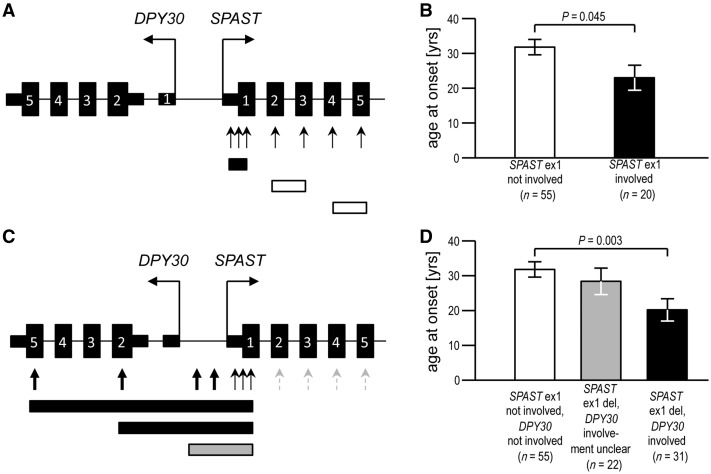
**Age at onset of symptoms is reduced in HSP patients with SPAST deletions that involve DPY30.** (**A**) MLPA probes in the standard commercially available assay (P165) target each *SPAST* exon with one or more probes (arrows), but do not analyse neighbouring regions/genes. Deletions that involve *SPAST* exon 1 (black box in the lower part of the figure) can be distinguished from deletions that spare *SPAST* exon 1 (white boxes as examples). (**B**) Age at onset in patients where *SPAST* exon 1 is part of the deletion is significantly younger than age at onset in patients with other *SPAST* deletions. (**C**) In a modified MLPA assay (P211), most probes against internal *SPAST* exons were removed (stippled grey arrows), and replaced by probes that target the adjacent gene *DPY30* or the intergenic region (bold arrows). This enabled classification of *SPAST* exon 1 deletion carriers into those in which *DPY30* was also at least partially deleted (examples shown by black boxes in the lower part of the panel), and those in which *DPY30* deletion status was inconclusive, as the deletion involved intergenic probes but no probes targeting the *DPY30* coding region (e.g. grey box). (**D**) Age at onset in patients with *SPAST* deletions, stratified on the status of whether the deletion also involved *DPY30*. Age at onset was significantly younger in patients where the *SPAST* deletion extended into *DPY30* coding exons, when compared to carriers of *SPAST* deletions that do not involve *SPAST* exon 1 (and, therefore, definitely spare *DPY30*). Histograms show mean ± SEM, *P*-values generated by two-tailed Student’s *t-*test.

We first set out to understand the discrepancy between the two initial reports that characterized the effect of *SPAST* exonic deletions on age at onset (Beetz *et al.*, 2006; Depienne *et al.*, 2007). We noticed an increased proportion of families with deletions involving exon 1 in the study that reported a younger age at onset [8 of 24 families (33%) in Depienne *et al.* (2007) versus 3 of 13 families (23%) in Beetz *et al.* (2006)]. This disproportion was even greater when numbers of patients with age at onset data (rather than numbers of families) were compared [16/44 or 36% with an exon 1 deletion in Depienne *et al.* (2007) versus 4/31 or 13% in Beetz *et al.* (2006)]. We reanalysed these data to test whether earlier age at onset was significantly related to involvement of *SPAST* exon 1. Indeed, mean age at onset for the 20 exon 1 deletion patients in both studies was 23.0 (±16.3) years, whereas it was 31.8 (±16.7) years for the 55 patients with deletions that did not involve exon 1 (Fig. 1A, B and Supplementary Table 1). Unfortunately any potential impact on severity or progression could not be analysed because of insufficient patient data. We concluded that *SPAST* exon 1 deletion is associated with a younger age at onset of HSP.

### Contiguous deletion of *DPY30* is associated with decreased age at onset in patients with *SPAST* exon 1 deletions

Considering that all *SPAST* exonic deletions (and indeed most, if not all, spastin mutations) almost certainly act via haploinsufficiency, the finding of clinical differences between families with the same mutational mechanism was puzzling and so we explored it further (Burger *et al.*, 2000; Fonknechten *et al.*, 2000; Beetz *et al.*, 2006). Of note, in a Japanese family a genomic deletion involving *SPAST* exons 1–4 extended into the adjacent gene *DPY30*, which is ubiquitously expressed, lies ∼24 kb upstream and is ‘head-to-head’ with *SPAST*, an arrangement that has been present in evolution since at least cartilaginous fish (Supplementary Fig. 1). This family had a young mean age at onset of 10.4 (±18.8) years (Miura *et al.*, 2011). We therefore hypothesized that reduced age at onset in patients with exon 1 *SPAST* deletions is explained by the involvement of *DPY30* in some families. To test this, we developed a novel MLPA assay in which several probes target the 5′ end of the *SPAST* gene, the *DPY30* gene, and the intergenic region (Fig. 1C). Moreover, we analysed additional families with deletions involving exon 1. Together with the families reported in the previous studies (Beetz *et al.*, 2006; Depienne *et al.*, 2007; Miura *et al.*, 2011), our cohort now included 21 families (53 patients) with *SPAST* exon 1 deletions, and 55 patients with *SPAST* deletions not involving exon 1 (Supplementary Table 2). In 10 of the families with *SPAST* exon 1 deletions (31 patients), the deleted region included at least the 5′UTR and exons 1 and 2 of *DPY30*, while for the remaining 11 families (22 patients), *DPY30* involvement was inconclusive (Fig. 1C). These genotypes were correlated with age at onset. Mean age at onset for carriers of deletions affecting both genes was significantly lower (at 20.2 ± 18.1 years) than in patients with *SPAST* deletions that spared *DPY30* (31.8 ± 16.7 years). Cases with inconclusive *DPY30* involvement, which likely represent a mixture of the former two classes, had an intermediate age at onset (28.5 ± 17.8 years) (Fig. 1D and Supplementary Table 2). The mean age at onset in the *SPAST*-only deletion patients is similar to that reported with conventional ‘small’ *SPAST* mutations (Chelban *et al.*, 2017). We concluded that the earlier age at onset observed in patients with *SPAST* exon deletions is accounted for by families where the deletion also involves *DPY30*.

### Reduced expression of *DPY30* does not affect *SPAST* transcription

We sought a mechanistic explanation for the apparent epistatic effect between *SPAST* and *DPY30*. The best characterized role of DPY30 is in transcriptional regulation via histone methylation as part of a multiprotein complex termed WRAD (WDR5, RBBP5, ASH2L and DPY30), which binds to SET1 family methytransferases and stimulates methylation of histone H3 at lysine 4 (Ernst and Vakoc, 2012). However, several non-canonical roles have also been described, including a role in the cytoplasm where it has been suggested to promote endosome-to-Golgi traffic, although this has not been directly tested (Xu *et al.*, 2009; Ali and Tyagi, 2017).

**Figure 2:**
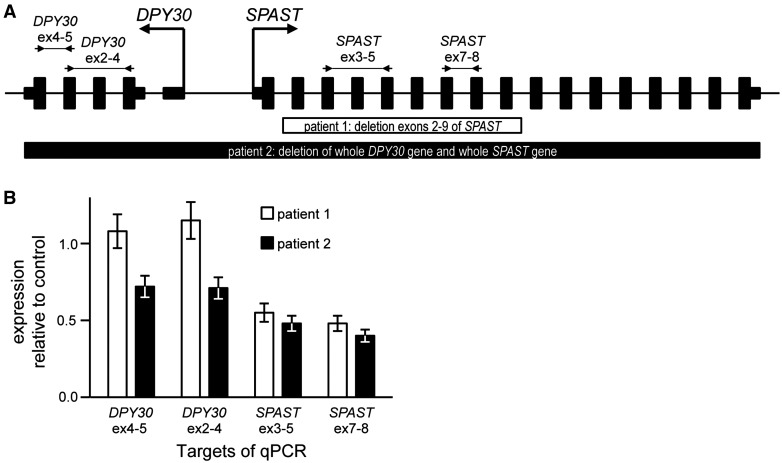
**DPY30 does not affect *SPAST* transcription.** (**A**) Fibroblast mRNA samples from a patient with a *SPAST*-only deletion (Patient 1, white box), from a patient with a *SPAST*+*DPY30* deletion (Patient 2, black box), and from a healthy control were analysed by exon-spanning qPCRs as indicated above the schematics of the genes. The expression levels (normalized to two housekeeping genes) in the patient samples relative to the control sample are shown in **B**. Values plotted are means ± SEM, *P*-values generated with paired two-tailed *t*-tests.

In view of the transcriptional role of DPY30, we examined whether haploinsufficiency of *DPY30* affects *SPAST* transcription. We performed quantitative RT-PCR experiments on RNA extracted from fibroblasts from (i) a control subject; (ii) an HSP patient with a deletion involving spastin only (exons 2–9; Patient 1); and (iii) a patient with a deletion involving *SPAST* and *DPY30* (*SPAST* exons 1–17 + *DPY30* exons 1–5; Patient 2), using primers amplifying two independent fragments of the *DPY30* or *SPAST* transcripts. The patients with the *SPAST*-only deletion and the *SPAST*+*DPY30* deletion had similar levels of *SPAST* transcript (∼40% of the transcript levels of the control) (Fig. 2A and B). We concluded that DPY30 does not significantly influence *SPAST* transcription.

### DPY30 regulates endosomal tubulation and endosome-to-Golgi traffic of M6PR

**Figure 3:**
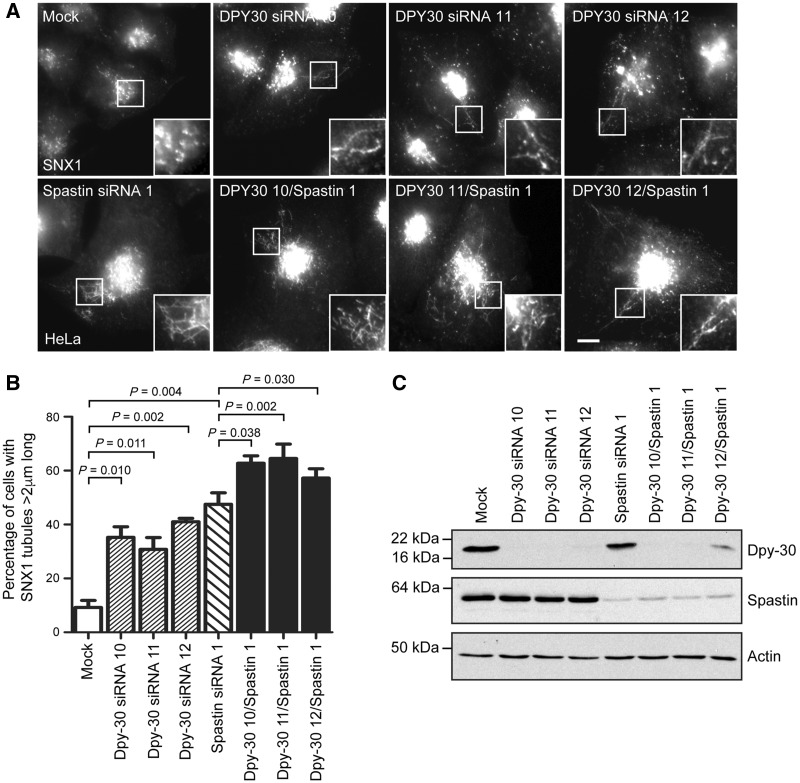
**DPY30 regulates endosomal tubulation and endosome-to-Golgi traffic.** (**A**) HeLa cells were transfected with the siRNA oligonucleotides indicated, then fixed and labelled for endogenous SNX1. In each experimental condition 100 cells were analysed and the percentage of cells with at least one SNX1 tubule >2 μm in length was reported. The mean percentage of cells with endosomal tubules in *n* = 4 biological repeats is shown in **B**. Corresponding immunoblots to verify protein depletion are shown in **C**. *P*-values were generated by paired two-tailed *t*-test, error bars show mean ± SEM. Scale bar = 10 μm in light micrographs.

As spastin regulates endosomal tubule fission and endosome-to-Golgi traffic, we next asked whether DPY30 influences these phenotypes. In cells lacking spastin, defective endosomal tubule fission causes an increase in the number of long tubules labelled by sorting nexin 1 (SNX1), a marker of tubules that are involved in endosome-to-Golgi traffic of M6PRs (Allison *et al.*, 2013, 2017). Interestingly, an increased proportion of cells lacking DPY30 developed long endosomal tubules compared to control cells (Fig. 3A–C). This effect was seen with three separate siRNA oligonucleotides, confirming that it was not due to an off-target effect of siRNA transfection. The size of the effect was not as large as observed in cells lacking spastin, but importantly we saw an additive effect of combined spastin and DPY30 depletion (Fig. 3A and B). We examined whether other members of the WRAD complex also regulate endosomal tubulation. While we found no significant effects with depletion of any individual complex member, in each case there was a non-significant trend towards increased endosomal tubulation (Supplementary Fig. 2). We therefore cannot exclude the idea that DPY30’s role in regulation of endosomal tubulation is as part of this complex.

**Figure 4:**
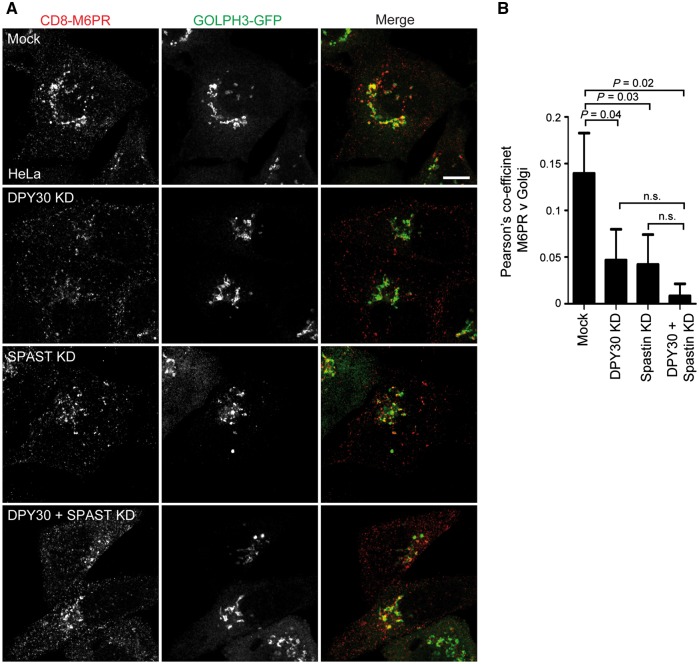
**DPY30 regulates endosome-to-Golgi traffic.** (**A**) Anti-CD8 antibody uptake experiments in HeLa cells stably expressing CD8-ciM6PR and GFP-GOLPH3 (a Golgi marker), and subjected to DPY30, spastin alone or spastin plus DPY30 depletion by siRNA knock-down (KD), using a pool of siRNAs to the proteins indicated. Cells were fixed 30 min after antibody uptake and CD8 and GFP signal visualized by immunofluorescence microscopy. Mean ± SEM co-localization between the markers (Pearson’s correlation) at 30 min is shown in the histogram **B** (*n* = 6). Scale bar = 10 μm. *P*-values calculated using paired one-tailed *t*-tests.

We next examined whether DPY30 affects endosome-to-Golgi traffic. A previous report described an abnormal distribution of M6PR in cells lacking DPY30, but did not directly examine endosome-to-Golgi traffic (Xu *et al.*, 2009). To test this, we employed an assay using a HeLa cell line stably expressing CD8-epitope tagged M6PR tail (Seaman, 2007). Some tagged protein is present at the plasma membrane and subject to endocytosis and subsequent trafficking to the trans Golgi network, so anti-CD8 antibody feeding experiments can be used to report on endosome-to-Golgi traffic of M6PR. In wild-type cells, we found strong co-localization between CD8-M6PR and the Golgi apparatus 30 min after antibody internalization. However, in cells lacking either spastin, DPY30 or both proteins, we saw a reduction in the amount of co-localization between CD8-M6PR and the Golgi 30 min after internalization, consistent with inhibition of endosome-to-Golgi traffic of the receptor (Fig. 4A and B). M6PR distribution in these cells tended to be more peripheral, consistent with the previous report (Xu *et al.*, 2009). There was a non-significant trend towards an additive effect on M6PR endosome-to-Golgi traffic in cells lacking both DPY30 and spastin. Similarly, using a probe (Magic Red-cathepsin B) that fluoresces on cleavage by the M6P-tagged lysosomal enzyme cathepsin B, we saw slight reductions in enzyme activity with depletion of DPY30 or spastin alone, with significant additive effects of combined depletion (Supplementary Fig. 3).

As cells lacking DPY30 have increased SNX1 endosomal tubulation accompanied by reduced endosome-to-Golgi traffic of cargo that normally traffics via SNX1 tubules, we concluded that, like spastin, DPY30 promotes fission of SNX1-labelled endosomal tubules. Thus DPY30 is a novel component of the machinery that drives endosomal tubule fission.

### Cells lacking DPY30 have abnormal lysosomal morphology

Cellular models of spastin-HSP, including primary neurons from a spastin-HSP mouse model and patient iPSC-derived neurons, develop enlarged lysosomes and a characteristic ultrastructural appearance. This abnormal ultrastructure affects lysosomes of all sizes and comprises accumulation of abnormal membrane material within the lysosome (Connell *et al.*, 2016; Allison *et al.*, 2017).

**Figure 5:**
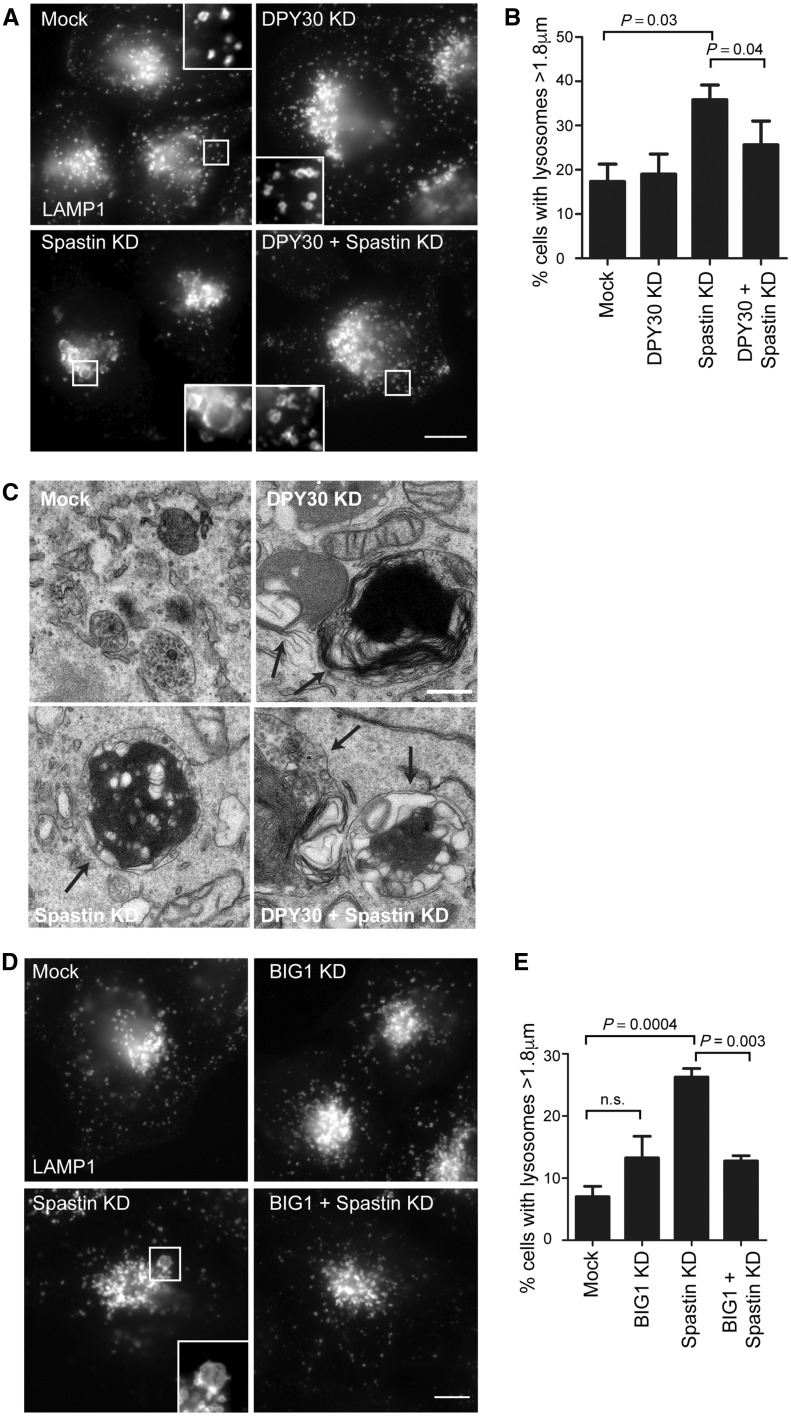
**DPY30 influences lysosomal morphology.** (**A**) HeLa cells were transfected with a pool of siRNAs targeting the proteins indicated, then fixed and imaged for endogenous LAMP1. The percentage of cells with lysosomes >1.8 μm (*n* = 100 cells per condition) was counted and the mean of six biological repeats is plotted in **B**. (**C**) HeLa cells were transfected with a pool of siRNAs targeting the proteins indicated, fixed and processed for electron microscopy. Lysosomes containing abnormal intralumenal content are indicated by arrows. (**D**) HeLa cells were transfected with a pool of siRNAs targeting the proteins indicated, then fixed and imaged for endogenous LAMP1. The percentage of cells with lysosomes >1.8 μm (*n* = 100 cells per condition) was counted and the mean of four biological repeats is plotted in **E**. *P*-values were generated by paired two-tailed *t*-test, error bars show mean ± SEM. Scale bar = 10 μm in light micrographs; 500 nm in electron micrographs.

Against this background, we examined whether DPY30 influences lysosomal size or ultrastructural morphology. HeLa cells depleted of DPY30 showed no increase in the mean size of the largest lysosome per cell or in the percentage of cells with lysosomes >1.8 µm diameter (Fig. 5A and B). However, by electron microscopy we observed the presence of highly abnormal lysosomes. These lysosomes contained accumulations of membrane material that could be arranged in loose tangles or in dense honeycomb networks, and which was strikingly similar to structures seen in cells lacking spastin (Fig. 5C) (Allison *et al.*, 2017). Lysosomes in cells lacking DPY30 or spastin showed minimal co-localization with the autophagic marker LC3, indicating that they are predominantly not autolysosomes (Supplementary Fig. 4).

We then examined whether DPY30 influences lysosomal phenotypes in cells lacking spastin. We examined lysosomal size, postulating that DPY30 and spastin depletion would have additive effects. Surprisingly we found that depletion of DPY30 in cells lacking spastin rescued the increase in the proportion of cells with enlarged lysosomes observed in cells lacking only spastin (Fig. 5A and B). We found a similar rescue effect following depletion of two other members of the WRAD complex, RBBP5 and Ash2L (Supplementary Fig. 5). However, the abnormal ultrastructural morphology seen in cells lacking spastin was not rescued by additional DPY30 depletion, as highly abnormal lysosomes were present in cells depleted of both proteins (Fig. 5C).

Lysosomes in cells lacking spastin become more acidic (Allison *et al.*, 2017). We examined lysosomal pH in cells lacking DPY30, and found them to be significantly more alkaline than in wild-type cells. Depletion of both DPY30 and spastin resulted in a mean lysosomal pH that was significantly lower than in wild-type cells, but higher than in spastin-only depleted cells (Supplementary Fig. 6). As it is not known whether the increased acidity in cells lacking spastin is a direct pathological mechanism or a homeostatic response in an attempt to clear the substrate accumulation within the lysosome, the functional consequences of this raised pH are unclear.

We concluded from these experiments that DPY30 acts in the same way as spastin to prevent accumulation of abnormal material in lysosomes, but has differing effects on the regulation of lysosomal size and pH.

### Depletion of the DPY30 binding partner BIG1 phenocopies DPY30 depletion

As haploinsufficiency of *DPY30* increases severity of the spastin-HSP phenotype by reducing age at onset, the rescue of spastin-depletion-induced lysosomal enlargement (although not lysosomal ultrastructural abnormalities) by depletion of DPY30 is at first sight counter-intuitive. We therefore explored a potential mechanism of this in more detail. DPY30 interacts with two guanine nucleotide exchange factor (GEF) proteins, ARFGEF1 and ARFGEF2 [also known as Brefeldin-inhibited GEFS 1 and 2 (BIG1 and BIG2)]. The simplest model for the functional relationship between these proteins is that DPY30 promotes the action of BIG1/2, as like DPY30, inhibition of BIG1/2 causes increased endosomal tubulation and blocks in endosome to Golgi traffic, suggestive of defective endosomal tubule fission. However, BIG1/2 also promote vesicular transport from the trans Golgi network to the endosomal pathway. (Ishizaki *et al.*, 2008; Manolea *et al.*, 2008; Xu *et al.*, 2009; Xia *et al.*, 2010; D’Souza *et al.*, 2014). Indeed, at steady state both cytoplasmic DPY30 and BIG1/2 predominantly localize to the trans Golgi network, and so inhibition of BIG1/2 caused by loss of DPY30 would be expected to reduce membrane traffic from the trans Golgi network to the endosomal pathway, as well as affecting endosomal tubule fission. We therefore predicted that depletion of a BIG protein would phenocopy the effects of DPY30 depletion on lysosomal size. We focused on BIG1, as DPY30 recruitment to the trans Golgi network requires this protein, and found that the increased lysosomal size phenotype found in cells lacking spastin was indeed rescued by depletion of BIG1 (Fig. 5D and E) (Xu *et al.*, 2009).

## Discussion

In this study we identify an epistatic interaction between *SPAST* and *DPY30* that influences age at onset in spastin-HSP. Mutations in *SPAST* account for up to 60% of familial HSP cases in northern Europe and North America, and of these spastin-HSP cases 10–20% may be caused by large rearrangements, predominantly deletions (Beetz *et al.*, 2006; Depienne *et al.*, 2007; Sulek *et al.*, 2013; Schule *et al.*, 2016; Chelban *et al.*, 2017). We now show that when these deletions also involve the adjacent gene *DPY30* [as happened in at least 20% of *SPAST* deletion cases reported in Beetz *et al.* (2006) and Depienne *et al.* (2007)], mean age at onset of HSP is significantly reduced. This provides an explanation for the previously perplexing observation that patients with *SPAST* whole exon deletions have a younger mean age at onset than patients with other mutational classes that are also expected to cause haploinsufficiency.

The *DPY30* gene deletions identified were contiguous with *SPAST* deletions, and almost certainly arose as part of the same mutational event in each family; 70% of *SPAST* copy number variants are caused by an Alu-repeat based mechanism, with the Alu-rich genomic architecture of *SPAST* (40% of the gene’s sequence are Alu elements) making it prone to these events (Boone *et al.*, 2014). Of note, *DPY30* has an even higher fraction of Alu-derived sequence (51%). However, in the general population copy number variants involving *DPY30* appear to be rare, with deletions having a frequency of 1 and duplications a frequency of 5 in 32 850 in non-Finnish European alleles (ExAC database) (Lek *et al.*, 2016). In addition, missense variants and variants predicted to result in possible loss of function via splicing effects are also individually and collectively rare in *DPY30*; the most common putative splice site mutation listed in ExAC has an allele frequency of <0.0003 (ExAC database) (Lek *et al.*, 2016). For this reason, we consider it unlikely that polymorphisms in *DPY30* will make a substantial contribution to influencing age at onset in patients with spastin-HSP not caused by exon deletions. In addition, in view of the relatively small effect of DPY30 depletion (which is almost complete by immunoblotting) versus spastin depletion on endosomal tubulation and M6PR traffic, we consider it unlikely that haploinsufficiency of *DPY30* could itself cause HSP. Knockout of *Dpy30* in mice causes embryonic lethality, and so it is again unlikely that recessive *DPY30* mutations cause HSP (Bertero *et al.*, 2015).

Variability in age at onset of neurogenetic diseases is often ascribed to effects of genetic background, i.e. the effects a gene or genes that modify the function of the disease gene. Such modifier genes are being systematically sought, through candidate gene or genome-wide efforts in disease populations (Mehta *et al.*, 2007; Gan-Or *et al.*, 2015; Pla-Martin *et al.*, 2015; Jiang *et al.*, 2016; Velez *et al.*, 2016). The mechanism of such effects could include participation of the encoded proteins in the same cell biological pathway or protein complex, or the existence of a regulatory relationship between the encoded proteins. However, there are very few examples where modifier genes have been identified in genetic neurodegenerative disease and where the underlying cell biological mechanism of the epistatic effect has also been characterized. Thus, by showing that DPY30, like spastin, regulates endosomal sorting of M6PR and consequently lysosomal morphology, we provide both a mechanistic explanation for an epistatic effect in a genetic neurodegenerative disease, and proof of the principle that this may be due to functional effects on the same pathway.

**Figure 6:**
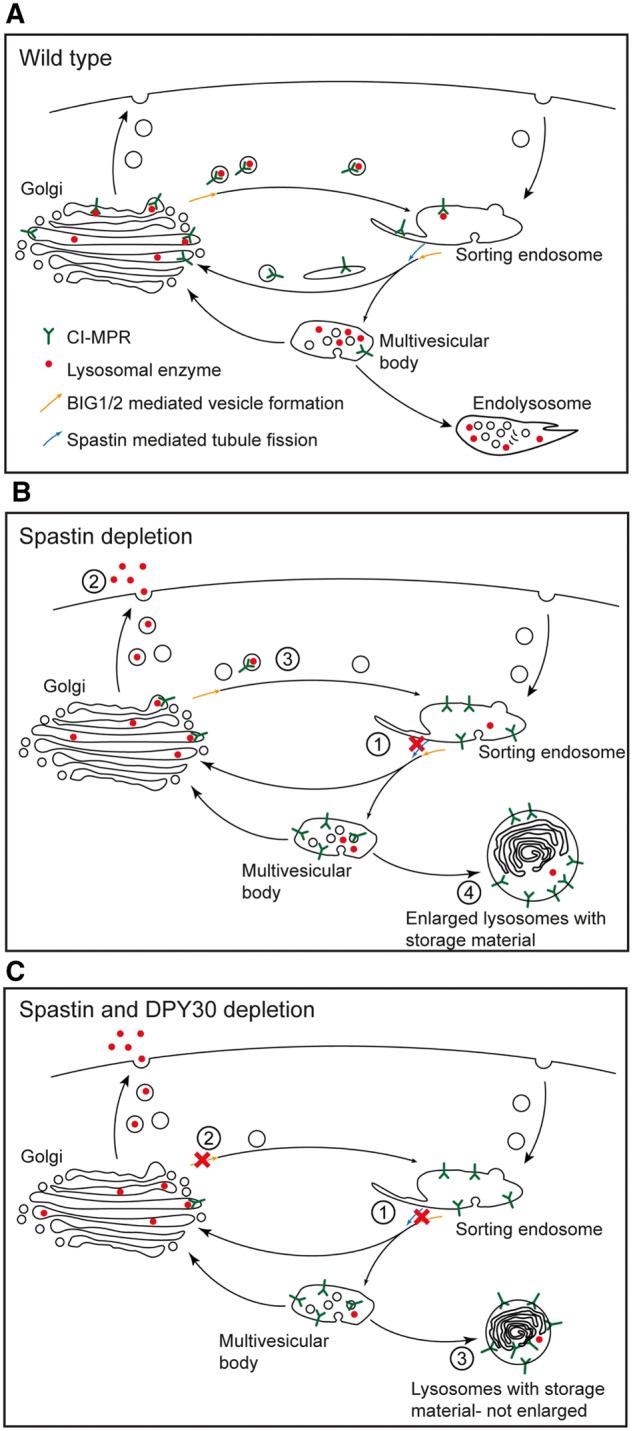
**Model of role of spastin and DPY30 in membrane traffic pathways, and how these might influence lysosomes when disrupted.** (**A**) In the normal situation, M6PR cycles between endosomes and the Golgi, capturing lysosomal enzymes at the Golgi for delivery back to the endolysosomal system. The sorting endosome matures into the multivesicular body and the accompanying acidification promotes dissociation of lysosomal enzymes from M6PR. During the maturation process, empty M6PR is sorted back to the Golgi in endosomal tubules that require spastin, DPY30 and likely BIG1/2 to break efficiently from the parent endosome. (**B**) In cells lacking spastin endosomal tubule fission is deficient (1), so M6PR traffic from the endosome-to-Golgi is inhibited and M6PR accumulates in the endolysosomal pathway. Lack of M6PR at the Golgi means that lysosomal enzymes are not captured, and instead are secreted (2) rather than delivered efficiently to the endolysosomal system (3). These effects result in the development of enlarged (through failure of membrane sorting away from the lysosomal pathway and substrate accumulation), morphologically abnormal (through substrate accumulation) lysosomes (4). (**C**) In cells lacking spastin and DPY30, traffic of M6PR from the endosomes to the Golgi is inhibited by inefficient endosomal tubule fission (1), but in addition DPY30 promotes BIG1/2-mediated traffic out of the Golgi, so lack of DPY30 inhibits Golgi to endosome traffic (2). Both effects reduce enzyme delivery to the lysosome, but will tend to have opposing impacts on lysosomal size. Thus in cells lacking both spastin and DPY30, lysosomal size depends on the balance between these two effects, but effects on enzyme delivery could be additive (3), especially in the case of haploinsufficiency of both.

We found a consistent trend towards increased endosomal tubulation when we depleted WRAD complex members other than DPY30, and we saw a similar rescue of the lysosomal size phenotype associated with lack of spastin when we depleted other WRAD components. These results are consistent with the findings that other WRAD complex members regulate M6PR localization, and suggest that the effect of DPY30 on membrane traffic pathways is as part of the WRAD complex, and presumably involves protein methylation (Xu *et al.*, 2009). The cytoplasmic targets of the WRAD complex, and the functional consequences of WRAD-mediated methylation of cytoplasmic proteins, are obscure. However, in general, methylation has been shown to influence several cytoplasmic biochemical pathways, for example, by altering binding affinities between pathway components, or regulating phosphorylation status (Biggar and Li, 2015). In view of their interaction with DPY30 and their known role in regulating endosomal tubulation and trans Golgi network to endosome vesicular traffic, we speculate that DPY30 and the WRAD complex may methylate BIG1/2, which could promote interaction and activation of their downstream targets such as the GTPase ARF1, to drive these processes (Gillingham and Munro, 2007; Ishizaki *et al.*, 2008; Manolea *et al.*, 2008; Xu *et al.*, 2009; Xia *et al.*, 2010; D’Souza *et al.*, 2014). Such a mechanism would be consistent with our finding that loss of BIG1 phenocopies the effect of loss of DPY30 in rescuing the increased lysosomal size in cells lacking spastin. Final lysosomal size in cells lacking spastin and DPY30 is likely to be governed by the balance of effects of loss of DPY30 in inhibiting endosomal tubule fission (which will tend to increase lysosomal size) or trans Golgi network to endosome traffic (which will tend to reduce lysosomal size) (Fig. 6). Importantly, although the size of the lysosomes is normalized in cells lacking both DPY30 and spastin, their ultrastructure was still highly abnormal. Indeed, we would expect that lack of DPY30 and spastin would have an additive effect on lysosomal functional abnormalities, as it would reduce traffic of enzymes to lysosomes by two mechanisms: (i) reduced capture of lysosomal enzymes because of reduced availability of M6PR at the trans Golgi network, due to defective SNX1 endosomal tubule fission; and (ii) defective BIG1-mediated vesicular transport from the trans Golgi network to the endolysosomal system, caused by lack of DPY30.

In summary, we have identified and delineated a cell biological mechanistic basis for an epistatic effect between DPY30 and spastin that influences age at onset in a significant subgroup of spastin-HSP patients. Our study supports the principle that alterations in genes that encode proteins functioning in the same cell biological pathway can explain epistatic effects in human disease in general, and neurodegenerative disease in particular. Furthermore, it also suggests a potential therapeutic strategy, as we predict that manipulations that increase DPY30 function could increase age at onset in spastin-HSP.

## Supplementary Material

Supplementary Figures

Supplementary Tables

Supplementary Figure 6

## Abbreviations

BIG1/2: brefeldin-inhibited GEFS 1/2
HSP: hereditary spastic paraplegia
MLPA: multiplex ligation-dependent probe amplification

## References

[awy034-B1] Ali A, Tyagi S Diverse roles of WDR5-RbBP5-ASH2L-DPY30 (WRAD) complex in the functions of the SET1 histone methyltransferase family. J Biosci 2017; 42: 155–9. 28229975 10.1007/s12038-017-9666-9

[awy034-B2] Allison R, Edgar JR, Pearson G, Rizo T, Newton T, Günther S, Defects in ER–endosome contacts impact lysosome function in hereditary spastic paraplegia. J Cell Biol 2017; 216: 1337–55. 28389476 10.1083/jcb.201609033 PMC5412567

[awy034-B3] Allison R, Lumb JH, Fassier C, Connell JW, Ten Martin D, Seaman MN, An ESCRT-spastin interaction promotes fission of recycling tubules from the endosome. J Cell Biol 2013; 202: 527–43. 23897888 10.1083/jcb.201211045 PMC3734076

[awy034-B4] Beetz C, Nygren AO, Schickel J, Auer-Grumbach M, Burk K, Heide G, High frequency of partial SPAST deletions in autosomal dominant hereditary spastic paraplegia. Neurology 2006; 67: 1926–30. 17035675 10.1212/01.wnl.0000244413.49258.f5

[awy034-B5] Bertero A, Madrigal P, Galli A, Hubner NC, Moreno I, Burks D, Activin/Nodal signaling and NANOG orchestrate human embryonic stem cell fate decisions by controlling the H3K4me3 chromatin mark. Genes Dev 2015; 29: 702–17. 25805847 10.1101/gad.255984.114 PMC4387713

[awy034-B6] Biggar KK, Li SSC Non-histone protein methylation as a regulator of cellular signalling and function. Nat Rev Mol Cell Biol 2015; 16: 5–17. 25491103 10.1038/nrm3915

[awy034-B7] Boone PM, Yuan B, Campbell IM, Scull JC, Withers MA, Baggett BC, The Alu-rich genomic architecture of SPAST predisposes to diverse and functionally distinct disease-associated CNV alleles. Am J Hum Genet 2014; 95: 143–61. 25065914 10.1016/j.ajhg.2014.06.014 PMC4129405

[awy034-B8] Burger J, Fonknechten N, Hoeltzenbein M, Neumann L, Bratanoff E, Hazan J, Hereditary spastic paraplegia caused by mutations in the SPG4 gene. Eur J Hum Genet 2000; 8: 771–6. 11039577 10.1038/sj.ejhg.5200528

[awy034-B9] Chelban V, Tucci A, Lynch DS, Polke JM, Santos L, Jonvik H, Truncating mutations in SPAST patients are associated with a high rate of psychiatric comorbidities in hereditary spastic paraplegia. J Neurol Neurosurg Psychiatry 2017; 88: 681–7. 28572275 10.1136/jnnp-2017-315796 PMC5537546

[awy034-B10] Connell JW, Allison R, Reid E Quantitative gait analysis using a motorized treadmill system sensitively detects motor abnormalities in mice expressing ATPase defective spastin. PLoS One 2016; 11: e0152413. 27019090 10.1371/journal.pone.0152413 PMC4809716

[awy034-B11] Connell JW, Lindon C, Luzio JP, Reid E Spastin couples microtubule severing to membrane traffic in completion of cytokinesis and secretion. Traffic 2009; 10: 42–56. 19000169 10.1111/j.1600-0854.2008.00847.x PMC2709849

[awy034-B12] D’Souza RS, Semus R, Billings EA, Meyer CB, Conger K, Casanova JE Rab4 orchestrates a small GTPase cascade for recruitment of adaptor proteins to early endosomes. Curr Biol 2014; 24: 1187–98. 24835460 10.1016/j.cub.2014.04.003 PMC4059052

[awy034-B13] Depienne C, Fedirko E, Forlani S, Cazeneuve C, Ribai P, Feki I, Exon deletions of SPG4 are a frequent cause of hereditary spastic paraplegia. J Med Genet 2007; 44: 281–4. 17098887 10.1136/jmg.2006.046425 PMC2598038

[awy034-B14] Ernst P, Vakoc CR WRAD: enabler of the SET1-family of H3K4 methyltransferases. Brief Funct Genomics 2012; 11: 217–26. 22652693 10.1093/bfgp/els017 PMC3388306

[awy034-B15] Errico A, Ballabio A, Rugarli EI Spastin, the protein mutated in autosomal dominant hereditary spastic paraplegia, is involved in microtubule dynamics. Hum Mol Genet 2002; 11: 153–63. 11809724 10.1093/hmg/11.2.153

[awy034-B16] Evans KJ, Gomes ER, Reisenweber SM, Gundersen GG, Lauring BP Linking axonal degeneration to microtubule remodeling by Spastin-mediated microtubule severing. J Cell Biol 2005; 168: 599–606. 15716377 10.1083/jcb.200409058 PMC2171748

[awy034-B17] Fink JK Hereditary spastic paraplegia. Curr Neurol Neurosci Rep 2006; 6: 65–76. 16469273 10.1007/s11910-996-0011-1

[awy034-B18] Fonknechten N, Mavel D, Byrne P, Davoine CS, Cruaud C, Bonsch D, Spectrum of SPG4 mutations in autosomal dominant spastic paraplegia. Hum Mol Genet 2000; 9: 637–44. 10699187 10.1093/hmg/9.4.637

[awy034-B19] Gan-Or Z, Amshalom I, Bar-Shira A, Gana-Weisz M, Mirelman A, Marder K, The Alzheimer disease BIN1 locus as a modifier of GBA-associated Parkinson disease. J Neurol 2015; 262: 2443–7. 26233692 10.1007/s00415-015-7868-3

[awy034-B20] Gillingham AK, Munro S The small G proteins of the Arf family and their regulators. Annu Rev Cell Dev Biol 2007; 23: 579–611. 17506703 10.1146/annurev.cellbio.23.090506.123209

[awy034-B21] Harding AE Hereditary spastic paraplegias. Semin Neurol 1993; 13: 333–6. 8146482 10.1055/s-2008-1041143

[awy034-B22] Hazan J, Fonknechten N, Mavel D, Paternotte C, Samson D, Artiguenave F, Spastin, a new AAA protein, is altered in the most frequent form of autosomal dominant spastic paraplegia. Nat Genet 1999; 23: 296–303. 10610178 10.1038/15472

[awy034-B23] Hensiek A, Kirker S, Reid E Diagnosis, investigation and management of hereditary spastic paraplegias in the era of next-generation sequencing. J Neurol 2015; 272: 1601–12. 10.1007/s00415-014-7598-y PMC4503825 25480570

[awy034-B24] Hirst J, Edgar JR, Esteves T, Darios F, Madeo M, Chang J, Loss of AP-5 results in accumulation of aberrant endolysosomes: defining a new type of lysosomal storage disease. Hum Mol Genet 2015; 24: 4984–96. 26085577 10.1093/hmg/ddv220 PMC4527494

[awy034-B25] Ishizaki R, Shin HW, Mitsuhashi H, Nakayama K Redundant roles of BIG2 and BIG1, guanine-nucleotide exchange factors for ADP-ribosylation factors in membrane traffic between the trans-Golgi network and endosomes. Mol Biol Cell 2008; 19: 2650–60. 18417613 10.1091/mbc.E07-10-1067 PMC2397321

[awy034-B26] Iwanaga H, Tsujino A, Shirabe S, Eguchi H, Fukushima N, Niikawa N, Large deletion involving the 5, Eguchi H, Fukushima N, Niikawa N BIG2 and BIG1, guanine-nucleotideant hereditary spastic paraplegia. Am J Med Genet 2005; 133: 13–17. 10.1002/ajmg.a.30510 15637712

[awy034-B27] Jiang P, Jin X, Peng Y, Wang M, Liu H, Liu X, The exome sequencing identified the mutation in YARS2 encoding the mitochondrial tyrosyl-tRNA synthetase as a nuclear modifier for the phenotypic manifestation of Leber's hereditary optic neuropathy-associated mitochondrial DNA mutation. Hum Mol Genet 2016; 25: 584–96. 26647310 10.1093/hmg/ddv498

[awy034-B28] Khundadze M, Kollmann K, Koch N, Biskup C, Nietzsche S, Zimmer G, A Hereditary spastic paraplegia mouse model supports a role of ZFYVE26/SPASTIZIN for the endolysosomal system. PLoS Genet 2013; 9: e1003988. 24367272 10.1371/journal.pgen.1003988 PMC3868532

[awy034-B29] Lek M, Karczewski KJ, Minikel EV, Samocha KE, Banks E, Fennell T, Analysis of protein-coding genetic variation in 60,706 humans. Nature 2016; 536: 285–91. 27535533 10.1038/nature19057 PMC5018207

[awy034-B30] Lindsey JC, Lusher ME, McDermott CJ, White KD, Reid E, Rubinsztein DC, Mutation analysis of the spastin gene (SPG4) in patients with hereditary spastic paraparesis. J Med Genet 2000; 37: 759–65. 11015453 10.1136/jmg.37.10.759 PMC1757167

[awy034-B31] Lumb JH, Connell JW, Allison R, Reid E The AAA ATPase spastin links microtubule severing to membrane modelling. Biochim Biophys Acta 2012; 1823: 192–7. 21888932 10.1016/j.bbamcr.2011.08.010

[awy034-B32] Manolea F, Claude A, Chun J, Rosas J, Melançon P Distinct functions for Arf guanine nucleotide exchange factors at the Golgi complex: GBF1 and BIGs are required for assembly and maintenance of the Golgi stack and trans-Golgi network, respectively. Mol Biol Cell 2008; 19: 523–35. 18003980 10.1091/mbc.E07-04-0394 PMC2230590

[awy034-B33] Mehta SG, Watts GD, Adamson JL, Hutton M, Umberger G, Xiong S, APOE is a potential modifier gene in an autosomal dominant form of frontotemporal dementia (IBMPFD). Genet Med 2007; 9: 9–13. 17224685 10.1097/gim.0b013e31802d830d

[awy034-B34] Miura S, Shibata H, Kida H, Noda K, Toyama T, Iwasaki N, Partial SPAST and DPY30 deletions in a Japanese spastic paraplegia type 4 family. Neurogenetics 2011; 12: 25–31. 20857310 10.1007/s10048-010-0260-7

[awy034-B35] Newton TM, Reid E An automated image analysis system to quantify endosomal tubulation. PLoS One 2016; 11: e0168294. 28006827 10.1371/journal.pone.0168294 PMC5179261

[awy034-B36] Orr HT, Zoghbi HY Trinucleotide repeat disorders. Annu Rev Neurosci 2007; 30: 575–621. 17417937 10.1146/annurev.neuro.29.051605.113042

[awy034-B37] Pfaffl MW A new mathematical model for relative quantification in real-time RT-PCR. Nucleic Acids Res 2001; 29: e45. 11328886 10.1093/nar/29.9.e45 PMC55695

[awy034-B38] Pla-Martin D, Calpena E, Lupo V, Marquez C, Rivas E, Sivera R, Junctophilin-1 is a modifier gene of GDAP1-related Charcot-Marie-Tooth disease. Hum Mol Genet 2015; 24: 213–29. 25168384 10.1093/hmg/ddu440

[awy034-B39] Reid E The hereditary spastic paraplegias. J Neurol 1999; 246: 995–1003. 10631629 10.1007/s004150050503

[awy034-B40] Renvoise B, Chang J, Singh R, Yonekawa S, FitzGibbon EJ, Mankodi A, Lysosomal abnormalities in hereditary spastic paraplegia types SPG15 and SPG11. Ann Clin Transl Neurol 2014; 1: 379–89. 24999486 10.1002/acn3.64 PMC4078876

[awy034-B41] Roll-Mecak A, Vale RD Structural basis of microtubule severing by the hereditary spastic paraplegia protein spastin. Nature 2008; 451: 363–7. 18202664 10.1038/nature06482 PMC2882799

[awy034-B42] Sauter S, Miterski B, Klimpe S, Bonsch D, Schols L, Visbeck A, Mutation analysis of the spastin gene (SPG4) in patients in Germany with autosomal dominant hereditary spastic paraplegia. Hum Mutat 2002; 20: 127–32. 12124993 10.1002/humu.10105

[awy034-B43] Schule R, Wiethoff S, Martus P, Karle KN, Otto S, Klebe S, Hereditary spastic paraplegia: clinicogenetic lessons from 608 patients. Ann Neurol 2016; 79: 646–58. 26856398 10.1002/ana.24611

[awy034-B44] Seaman MN Cargo-selective endosomal sorting for retrieval to the Golgi requires retromer. J Cell Biol 2004; 165: 111–22. 15078902 10.1083/jcb.200312034 PMC2172078

[awy034-B45] Seaman MN Identification of a novel conserved sorting motif required for retromer-mediated endosome-to-TGN retrieval. J Cell Sci 2007; 120 (Pt 14): 2378–89. 17606993 10.1242/jcs.009654

[awy034-B46] Shoukier M, Neesen J, Sauter SM, Argyriou L, Doerwald N, Pantakani DV, Expansion of mutation spectrum, determination of mutation cluster regions and predictive structural classification of SPAST mutations in hereditary spastic paraplegia. Eur J Hum Genet 2009; 17: 187–94. 18701882 10.1038/ejhg.2008.147 PMC2986068

[awy034-B47] Sulek A, Elert E, Rajkiewicz M, Zdzienicka E, Stepniak I, Krysa W, Screening for the hereditary spastic paraplaegias SPG4 and SPG3A with the multiplex ligation-dependent probe amplification technique in a large population of affected individuals. Neurological Sciences 2013; 34: 239–42. 22203332 10.1007/s10072-011-0899-3

[awy034-B48] Svenson IK, Kloos MT, Gaskell PC, Nance MA, Garbern JY, Hisanaga S, Intragenic modifiers of hereditary spastic paraplegia due to spastin gene mutations. Neurogenetics 2004; 5: 157–64. 15248095 10.1007/s10048-004-0186-z

[awy034-B49] Varga RE, Khundadze M, Damme M, Nietzsche S, Hoffmann B, Stauber T, *In Vivo* evidence for lysosome depletion and impaired autophagic clearance in hereditary spastic paraplegia type SPG11. PLoS Genet 2015; 11: e1005454. 26284655 10.1371/journal.pgen.1005454 PMC4540459

[awy034-B50] Velez JI, Rivera D, Mastronardi CA, Patel HR, Tobon C, Villegas A, A mutation in DAOA modifies the age of onset in PSEN1 E280A Alzheimer's disease. Neural Plast 2016; 2016: 9760314. 26949549 10.1155/2016/9760314 PMC4753688

[awy034-B51] Xia B, Joubert A, Groves B, Vo K, Ashraf D, Djavaherian D, Modulation of cell adhesion and migration by the histone methyltransferase subunit mDpy-30 and its interacting proteins. PLoS One 2010; 5: e11771. 20668708 10.1371/journal.pone.0011771 PMC2909266

[awy034-B52] Xu Z, Gong Q, Xia B, Groves B, Zimmermann M, Mugler C, A role of histone H3 lysine 4 methyltransferase components in endosomal trafficking. J Cell Biol 2009; 186: 343–53. 19651892 10.1083/jcb.200902146 PMC2728403

[awy034-B53] Yip AG, Durr A, Marchuk DA, Ashley-Koch A, Hentati A, Rubinsztein DC, Meta-analysis of age at onset in spastin-associated hereditary spastic paraplegia provides no evidence for a correlation with mutational class. J Med Genet 2003; 40: e106. 12960222 10.1136/jmg.40.9.e106 PMC1735583

