# Supplementary material for: Mechanistic basis of an epistatic interaction reducing age at onset in hereditary spastic paraplegia
Source: Brain. 2018 Feb 22;141(5):1286–99. doi: 10.1093/brain/awy034 (PMC5917785; doi:10.1093/brain/awy034)
Supplement: Supplementary Figures [file awy034_brain-2017-01568-file008.pdf]

Supplementary Figure Legends

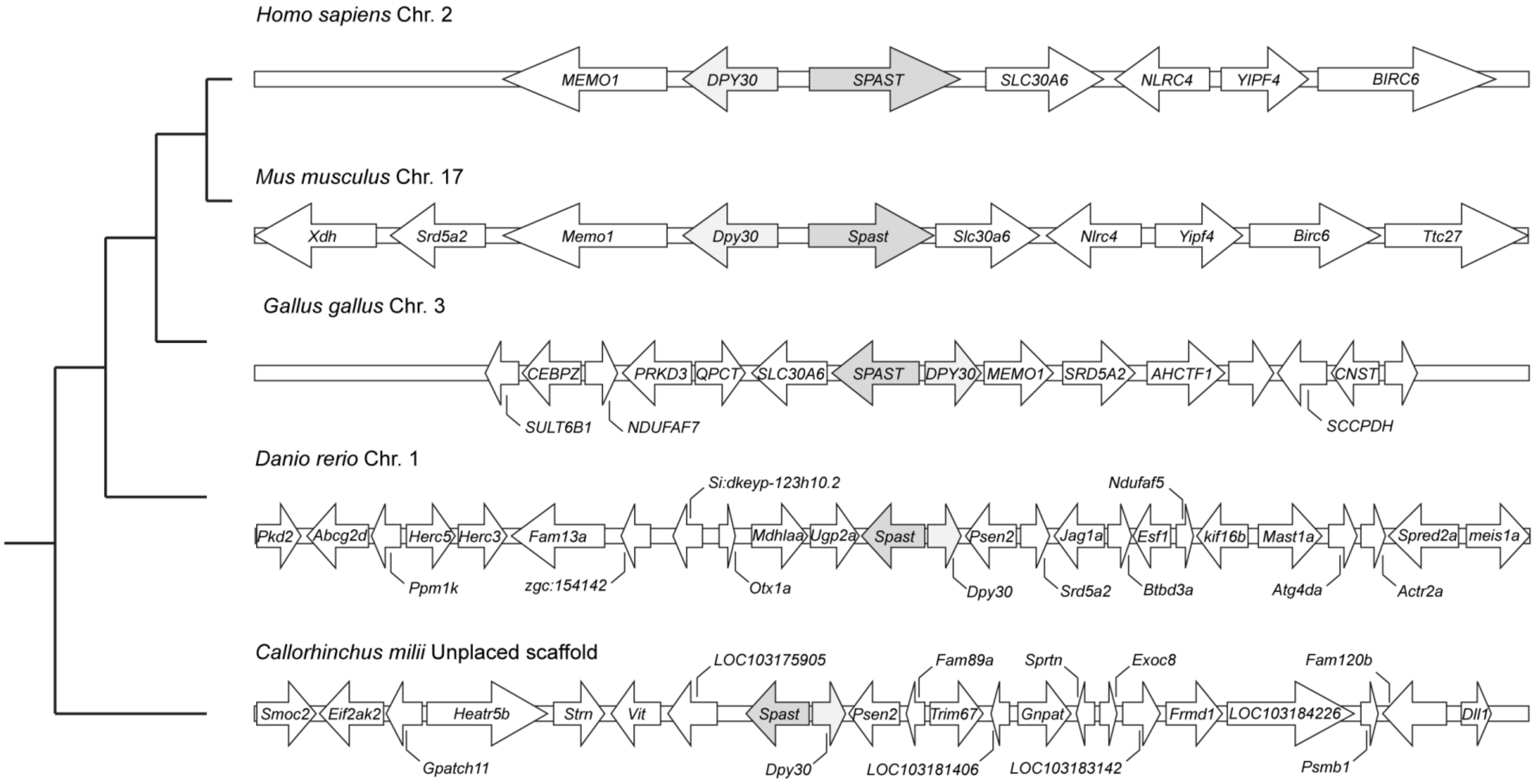

Supplementary Figure 1

Supplementary Figure 1. Evolutionary conservation of genomic relationship between *SPAST* and *DPY30*. Genomic architecture of the region containing *SPAST* and *DPY30* in selected species.

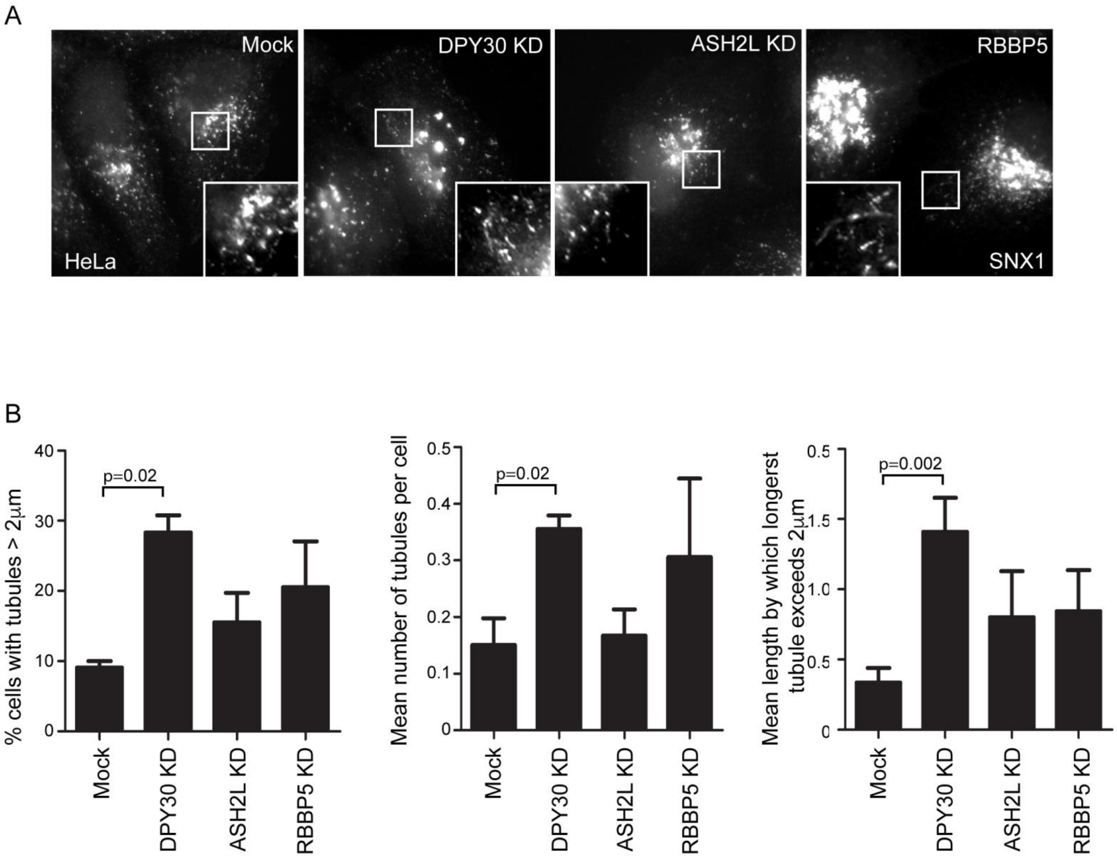

Supplementary Figure 2

Supplementary Figure 2. Quantification of SNX1 tubulation in cells lacking WRAD complex members. A) Hela cells were transfected with the siRNAs indicated, then fixed and imaged for endogenous SNX1. SNX1 tubulation was quantified using an automated image analysis system (Newton and Reid, 2016), and the results are presented in B) (n=4 biological repeats). P-values were generated by paired two-tailed t-test, bars show mean ± S.E.M. See Supplementary Fig. 5 for typical knock-down efficiency.

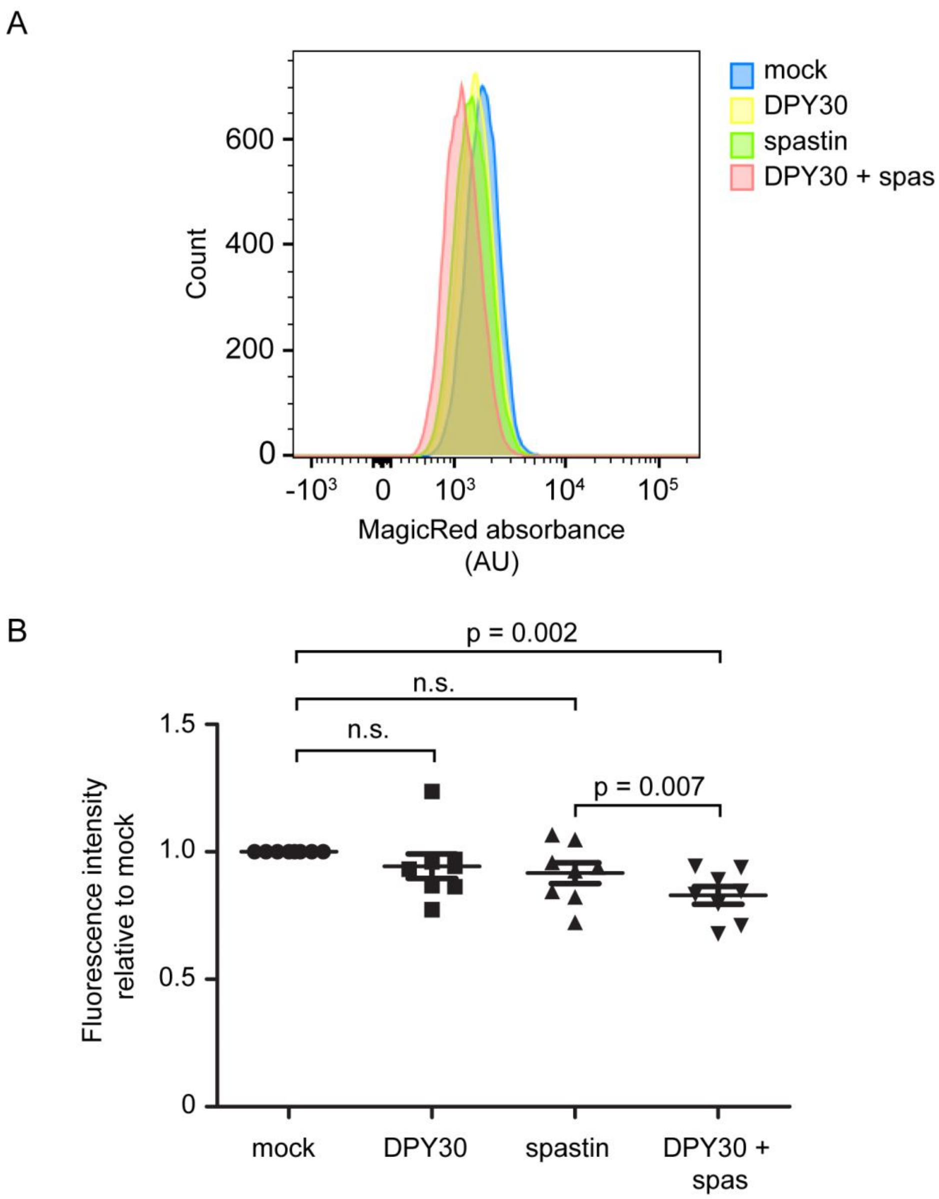

Supplementary Figure 3

Supplementary Figure 3. Cathepsin B enzyme activity in cells lacking DPY30, spastin or both proteins. Hela cells were incubated for an hour in the presence of Magic red-Cathepsin B, a marker whose fluorescence is activated by cleavage with Cathpsin B. Magic red fluorescence was then quantified in 20,000 cells per condition by fluorescence activated cell sorting analysis. A representative experiment is shown in A), and the mean fluorescence ± S.E.M from 9 such experiments are plotted in B). P-values were generated by paired two-tailed t-tests.

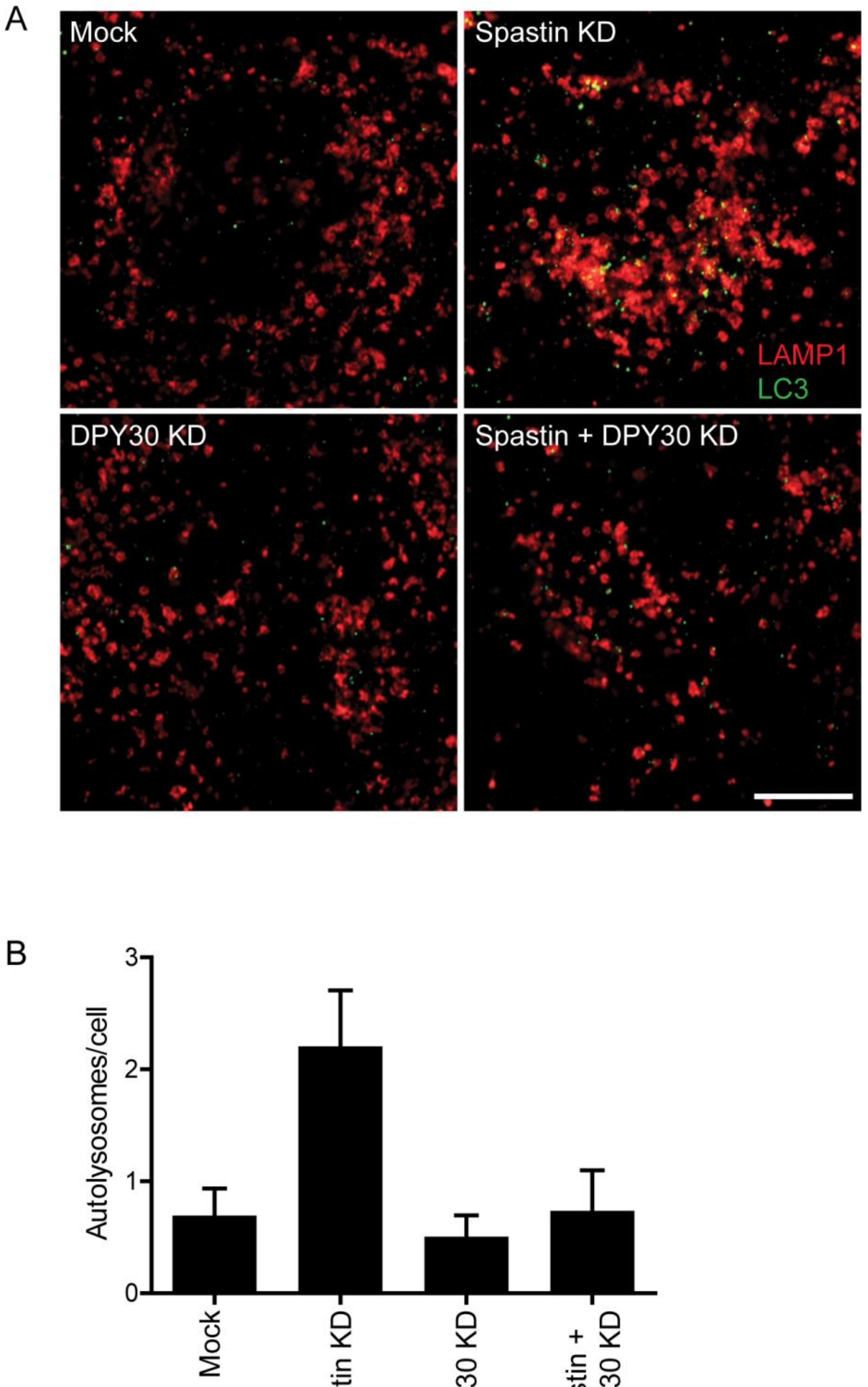

A
Mock
Spastin KD
LAMP1
LC3
DPY30 KD
Spastin + DPY30 KD
B
Autolysosomes/cell
3
2
1
0
Mock
Spastin KD
DPY30 KD
Spastin +
DPY30 KD

Supplementary Figure 4. Quantification of autolysosome numbers in cells lacking DPY30, spastin or both proteins. A) HeLa cells were transfected with the siRNAs indicated, then fixed and imaged for the lysosomal marker LAMP1 and the autophagosome marker LC3. Mean ± S.E.M number of autolysosomes (defined as LAMP1 structures that were also labelled with LC3) per cell was quantified in the corresponding histogram (n=3 experiments) (B). There were no statistically significant differences between the numbers of autolysosomes in any experimental condition. Scale bar in micrograph=10μm.

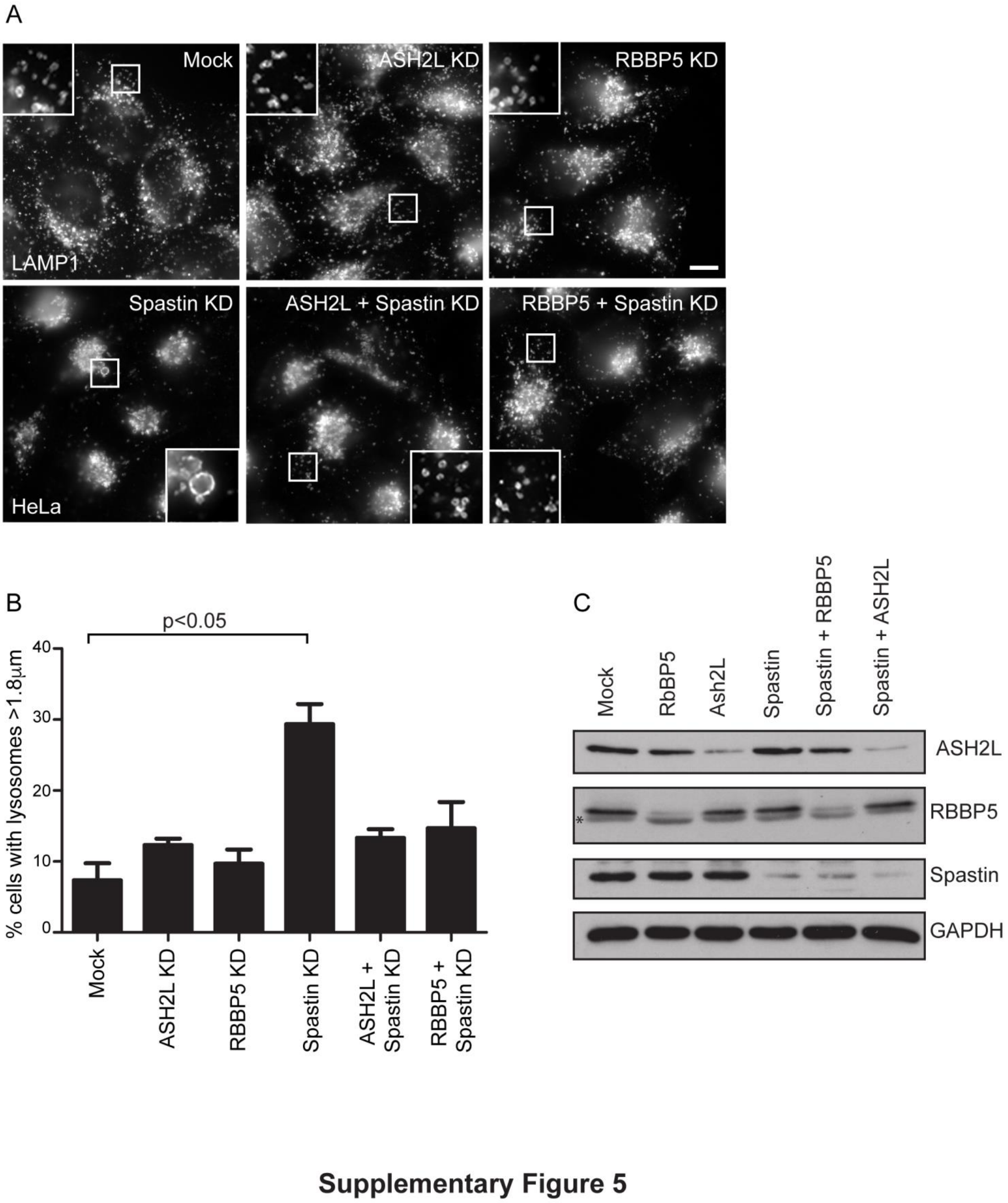

Supplementary Figure 5. WRAD complex member depletion rescues lysosomal size enlargement caused by lack of spastin. A) Hela cells were subject to siRNA knock down (KD) with pooled oligonucleotides targeting the proteins indicated, then fixed and imaged for endogenous LAMP1. The percentage of cells with lysosomes >1.8μm (n=100 cells per condition) and the mean results of 3 biological repeats are plotted in B). p-values were generated by paired two-tailed t-test, bars show mean ± S.E.M. C) Protein depletion was verified by immunoblotting. GAPDH blotting serves to verify equal protein loading between lanes. Scale bar in micrographs=10μm.

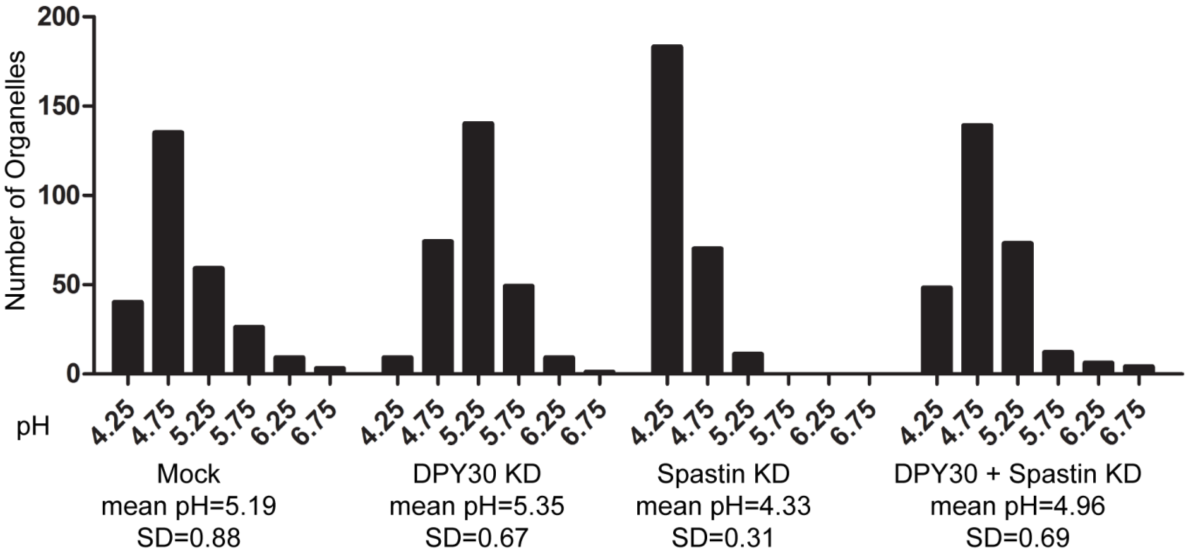

p-values

	DPY30	Spastin	DPY30+Spast
Mock	0.01	<0.0001	0.0003
DPY30		0.0001	<0.0001
Spastin			<0.0001

Supplementary Figure 6

Supplementary Figure 6. Lysosomal pH in cells lacking DPY30, spastin or both proteins. HeLa cells were transfected with the siRNAs indicated, then lysosomal pH was quantified using a ratiometric fluorescent microscopic method in which separate pH-quenchable and pH-insentitive fluorescently labelled dextrans were taken up into the terminal degradative (lysosomal) compartment, as described in the Methods. The pH distributions of individual puncta are shown in the histograms, while the p-values for differences between conditions are shown below (unpaired two-tailed t-test). The data for mock and spastin depletion have previously been presented in (Allison *et al.*, 2017)
