# Supplementary material for: Mechanistic basis of an epistatic interaction reducing age at onset in hereditary spastic paraplegia
Source: Brain. 2018 Feb 22;141(5):1286–99. doi: 10.1093/brain/awy034 (PMC5917785; doi:10.1093/brain/awy034)
Supplement: Supplementary Tables [file awy034_brain-2017-01568-file009.pdf]

Supplementary Tables

Supplementary Table 1. HSP-patients with large *SPAST* deletions reported in (Beetz *et al.*, 2006; Depienne *et al.*, 2007), and for which ages at onset were available.

Study	family	exons deleted	exon 1 involved	age at onset [yrs]
Beetz et al., 2006	B1	1	yes	34
Beetz et al., 2006	B2	1 to 3	yes	1
Beetz et al., 2006	B2	1 to 3	yes	16
Beetz et al., 2006	B3	1 to 7	yes	21
Depienne et al., 2007	165	1	yes	31
Depienne et al., 2007	172	1	yes	2
Depienne et al., 2007	172	1	yes	2
Depienne et al., 2007	172	1	yes	30
Depienne et al., 2007	366	1	yes	10
Depienne et al., 2007	366	1	yes	37
Depienne et al., 2007	366	1	yes	47
Depienne et al., 2007	366	1	yes	50
Depienne et al., 2007	366	1	yes	50
Depienne et al., 2007	774	1 to 4	yes	1
Depienne et al., 2007	628	1 to 17	yes	14
Depienne et al., 2007	202	1 to 17	yes	26
Depienne et al., 2007	253	1 to 17	yes	10
Depienne et al., 2007	253	1 to 17	yes	14
Depienne et al., 2007	253	1 to 17	yes	35
Depienne et al., 2007	282	1 to 17	yes	29
Beetz et al., 2006	B4	2 to 5	no	20
Beetz et al., 2006	B5	5 to 7	no	12
Beetz et al., 2006	B5	5 to 7	no	20
Beetz et al., 2006	B5	5 to 7	no	25
Beetz et al., 2006	B5	5 to 7	no	28
Beetz et al., 2006	B5	5 to 7	no	38
Beetz et al., 2006	B5	5 to 7	no	38
Beetz et al., 2006	B5	5 to 7	no	45
Beetz et al., 2006	B5	5 to 7	no	54
Beetz et al., 2006	B5	5 to 7	no	60
Beetz et al., 2006	B6	5 to 15	no	5
Beetz et al., 2006	B6	5 to 15	no	21
Beetz et al., 2006	B6	5 to 15	no	25
Beetz et al., 2006	B6	5 to 15	no	40
Beetz et al., 2006	B7	5 to 15	no	1
Beetz et al., 2006	B7	5 to 15	no	35
Beetz et al., 2006	B8	6 to 17	no	40
Beetz et al., 2006	B9	10	no	24
Beetz et al., 2006	B10	10 to12	no	53
Beetz et al., 2006	B11	13 to 16	no	9
Beetz et al., 2006	B11	13 to 16	no	40
Beetz et al., 2006	B11	13 to 16	no	50
Beetz et al., 2006	B11	13 to 16	no	60
Beetz et al., 2006	B12	16 to 17	no	30
Beetz et al., 2006	B12	16 to 17	no	43
Beetz et al., 2006	B13	17	no	20
Beetz et al., 2006	B13	17	no	40
Depienne et al., 2007	237	4 to 17	no	38
Depienne et al., 2007	742	5 to 6	no	40

Depienne et al., 2007	530	5 to 7	no	40
Depienne et al., 2007	530	5 to 7	no	77
Depienne et al., 2007	167	6	no	1
Depienne et al., 2007	167	6	no	15
Depienne et al., 2007	374	8 to12	no	37
Depienne et al., 2007	629	8 to 17	no	40
Depienne et al., 2007	152	8 to 17	no	25
Depienne et al., 2007	152	8 to 17	no	30
Depienne et al., 2007	152	8 to 17	no	32
Depienne et al., 2007	152	8 to 17	no	52
Depienne et al., 2007	157	9	no	17
Depienne et al., 2007	235	9 to 12	no	40
Depienne et al., 2007	335	10 to 16	no	33
Depienne et al., 2007	12	13	no	14
Depienne et al., 2007	12	13	no	18
Depienne et al., 2007	12	13	no	25
Depienne et al., 2007	12	13	no	54
Depienne et al., 2007	333	13	no	1
Depienne et al., 2007	333	13	no	26
Depienne et al., 2007	236	16	no	1
Depienne et al., 2007	236	16	no	53
Depienne et al., 2007	803	16	no	20
Depienne et al., 2007	803	16	no	36
Depienne et al., 2007	193	16 to 17	no	30
Depienne et al., 2007	168	17	no	30
Depienne et al., 2007	168	17	no	50

Supplementary Table 2. HSP-patients with large *SPAST* deletions and for which involvement of *DPY30* was analysed by MLPA (see also Fig. 1C,D).

**study**	**family**	**exons deleted**	***DPY30* involved**	**age at onset [yrs]**
Beetz et al., 2006	B4	2 to 5	no	20
Beetz et al., 2006	B5	5 to 7	no	12
Beetz et al., 2006	B5	5 to 7	no	20
Beetz et al., 2006	B5	5 to 7	no	25
Beetz et al., 2006	B5	5 to 7	no	28
Beetz et al., 2006	B5	5 to 7	no	38
Beetz et al., 2006	B5	5 to 7	no	38
Beetz et al., 2006	B5	5 to 7	no	45
Beetz et al., 2006	B5	5 to 7	no	54
Beetz et al., 2006	B5	5 to 7	no	60
Beetz et al., 2006	B6	5 to 15	no	5
Beetz et al., 2006	B6	5 to 15	no	21
Beetz et al., 2006	B6	5 to 15	no	25
Beetz et al., 2006	B6	5 to 15	no	40
Beetz et al., 2006	B7	5 to 15	no	1
Beetz et al., 2006	B7	5 to 15	no	35
Beetz et al., 2006	B8	6 to 17	no	40
Beetz et al., 2006	B9	10	no	24
Beetz et al., 2006	B10	10 to12	no	53
Beetz et al., 2006	B11	13 to 16	no	9
Beetz et al., 2006	B11	13 to 16	no	40
Beetz et al., 2006	B11	13 to 16	no	50
Beetz et al., 2006	B11	13 to 16	no	60
Beetz et al., 2006	B12	16 to 17	no	30
Beetz et al., 2006	B12	16 to 17	no	43
Beetz et al., 2006	B13	17	no	20
Beetz et al., 2006	B13	17	no	40
Depienne et al., 2007	237	4 to 17	no	38
Depienne et al., 2007	742	5 to 6	no	40
Depienne et al., 2007	530	5 to 7	no	40
Depienne et al., 2007	530	5 to 7	no	77
Depienne et al., 2007	167	6	no	1
Depienne et al., 2007	167	6	no	15
Depienne et al., 2007	374	8 to12	no	37
Depienne et al., 2007	629	8 to 17	no	40
Depienne et al., 2007	152	8 to 17	no	25
Depienne et al., 2007	152	8 to 17	no	30
Depienne et al., 2007	152	8 to 17	no	32
Depienne et al., 2007	152	8 to 17	no	52
Depienne et al., 2007	157	9	no	17
Depienne et al., 2007	235	9 to 12	no	40
Depienne et al., 2007	335	10 to 16	no	33
Depienne et al., 2007	12	13	no	14
Depienne et al., 2007	12	13	no	18
Depienne et al., 2007	12	13	no	25
Depienne et al., 2007	12	13	no	54

Depienne et al., 2007	333	13	no	1
Depienne et al., 2007	333	13	no	26
Depienne et al., 2007	236	16	no	1
Depienne et al., 2007	236	16	no	53
Depienne et al., 2007	803	16	no	20
Depienne et al., 2007	803	16	no	36
Depienne et al., 2007	193	16 to 17	no	30
Depienne et al., 2007	168	17	no	30
Depienne et al., 2007	168	17	no	50
this study	fam 578	1	?	32
this study	fam 884	1	?	39
this study	SAL 561	1	?	25
this study	SAL 561	1	?	50
Beetz et al., 2006	HIH10693	1	?	34
this study	5532A	1	?	5
this study	5532A	1	?	50
this study	5532A	1	?	65
this study	022722U	1	?	45
Beetz et al., 2007	CAM 23	1	?	3
Beetz et al., 2007	CAM 23	1	?	3
Beetz et al., 2007	CAM 23	1	?	3
Beetz et al., 2007	CAM 23	1	?	41
Beetz et al., 2006	LS616	1 to 3	?	1
Beetz et al., 2006	LS616	1 to 3	?	16
Beetz et al., 2006	LS616	1 to 3	?	16
this study	CAM 21	1 to 4	?	30
this study	CAM 21	1 to 4	?	30
this study	CAM 21	1 to 4	?	35
this study	CAM 21	1 to 4	?	36
this study	CAM 21	1 to 4	?	41
Depienne et al., 2007	BOR 202	1 to 17	?	26
Depienne et al., 2007	BOR 165	1	yes	31
Depienne et al., 2007	SAL 172	1	yes	2
Depienne et al., 2007	SAL 173	1	yes	2
Depienne et al., 2007	SAL 174	1	yes	30
Depienne et al., 2007	TAR 366	1	yes	10
Depienne et al., 2007	TAR 367	1	yes	37
Depienne et al., 2007	TAR 368	1	yes	47
Depienne et al., 2007	TAR 369	1	yes	50
Depienne et al., 2007	TAR 370	1	yes	50
Depienne et al., 2007	SAL 774	1 to 4	yes	1
Beetz et al., 2006	HIH10539	1 to 7	yes	21
Depienne et al., 2007	SAL 628	1 to 17	yes	14
Depienne et al., 2007	ROU 253	1 to 17	yes	10
Depienne et al., 2007	ROU 253	1 to 17	yes	14
Depienne et al., 2007	ROU 253	1 to 17	yes	35
Depienne et al., 2007	SAL 282	1 to 17	yes	29
this study	SAL 635	1 to 17	yes	12
this study	SAL 635	1 to 17	yes	25

this study	SAL 635	1 to 17	yes	40
this study	fam 1247-1267	1 to 17	yes	10
this study	fam 1247-1267	1 to 17	yes	12
this study	fam 1247-1267	1 to 17	yes	50
Miura et al. 2011	Miura family	1 to 4	yes	1
Miura et al. 2011	Miura family	1 to 4	yes	1
Miura et al. 2011	Miura family	1 to 4	yes	1
Miura et al. 2011	Miura family	1 to 4	yes	40
Miura et al. 2011	Miura family	1 to 4	yes	47
Miura et al. 2011	Miura family	1 to 4	yes	1
Miura et al. 2011	Miura family	1 to 4	yes	1
Miura et al. 2011	Miura family	1 to 4	yes	1
Miura et al. 2011	Miura family	1 to 4	yes	1
