# Supplementary figures and images for: Mechanistic basis of an epistatic interaction reducing age at onset in hereditary spastic paraplegia
Source: Brain. 2018 Feb 22;141(5):1286–99. doi: 10.1093/brain/awy034 (PMC5917785; doi:10.1093/brain/awy034)

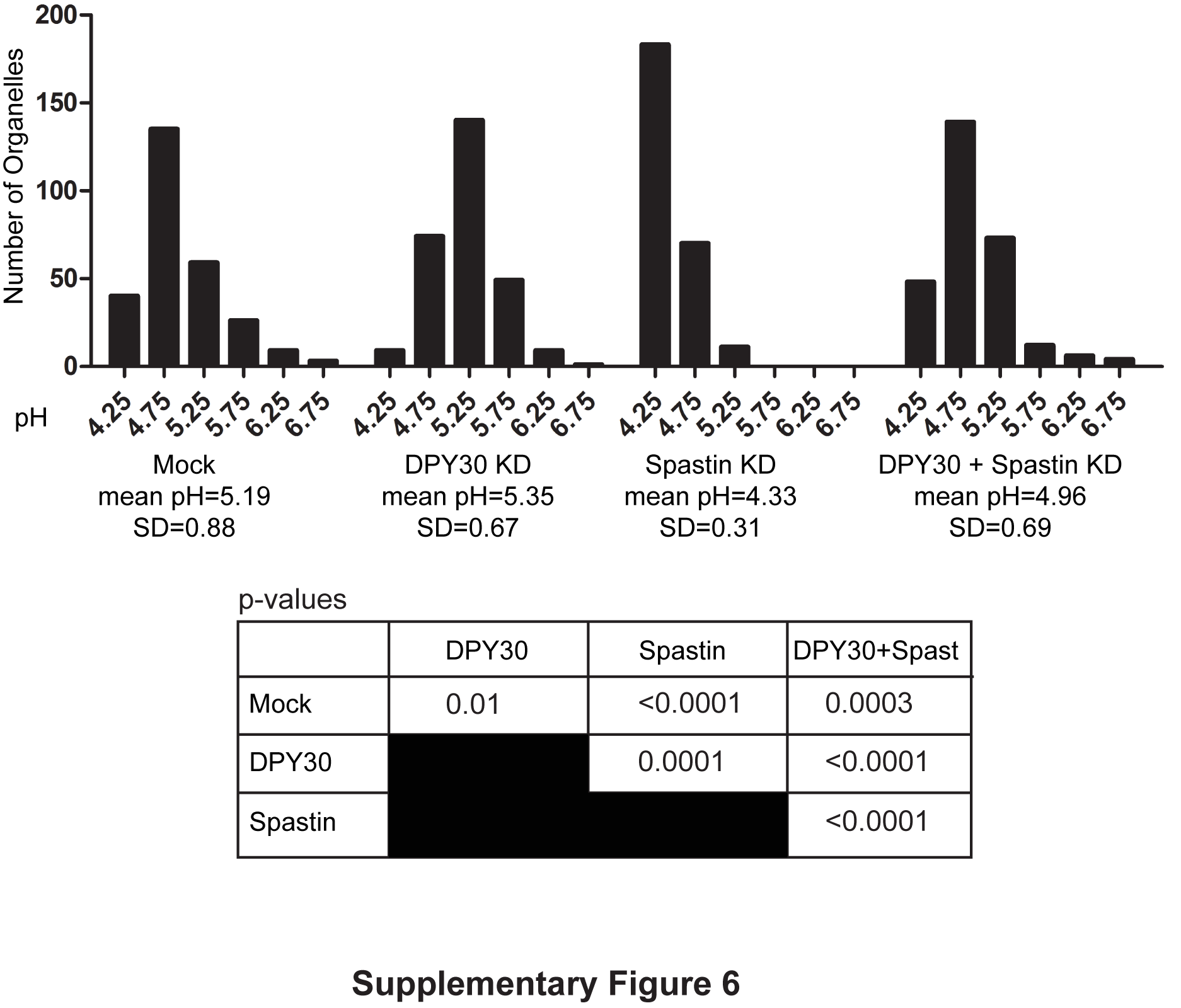

Supplement: Supplementary Figure 6 [file awy034_brain-2017-01568-file010.png]
